# Impact of sleep deprivation on colon cancer: Unraveling the KynA-P4HA2-HIF-1α axis in tumor lipid metabolism and metastasis

**DOI:** 10.1016/j.molmet.2025.102109

**Published:** 2025-02-06

**Authors:** Zuojie Peng, Jia Song, Wenzhong Zhu, Haijun Bao, Yuan Hu, Yongping Shi, Xukai Cheng, Mi Jiang, Feifei Fang, Jinhuang Chen, Xiaogang Shu

**Affiliations:** 1 Department of Gastrointestinal Surgery, Union Hospital, Tongji Medical College, Huazhong University of Science and Technology, Jiefang Road No.1277, Wuhan 430022, Hubei, China; 2 Department of Emergency Surgery, Union Hospital, Tongji Medical College, Huazhong University of Science and Technology, Jiefang Road No.1277, Wuhan 430022, Hubei, China

**Keywords:** Sleep deprivation, Colon cancer, Kynurenic acid, Liver metastasis, Lipid metabolism

## Abstract

**Objective:**

There is growing evidence that sleep deprivation promotes cancer progression. In addition, colon cancer patients often experience sleep deprivation due to factors such as cancer pain and side effects of treatment. The occurrence of liver metastases is an important factor in the mortality of colon cancer patients. However, the relationship between sleep deprivation and liver metastases from colon cancer has not been elucidated.

**Methods:**

A sleep deprivation liver metastasis model was constructed to evaluate the effect of sleep deprivation on liver metastasis of colon cancer. Subsequently, mice feces were collected for untargeted metabolomics to screen and identify the key mediator, Kynurenic acid (KynA). Furthermore, HILPDA was screened by transcriptomics, and its potential mechanism was explored through ChIP, co-IP, ubiquitination experiments, phenotyping experiments, etc.

**Results:**

Sleep deprivation promotes liver metastases in colon cancer. Functionally, sleep deprivation aggravates lipid accumulation and decreases the production of the microbiota metabolite KynA. In contrast, KynA inhibited colon cancer progression *in vitro*. *In vivo*, KynA supplementation reversed the promoting effects of sleep deprivation on liver metastases from colon cancer. Mechanistically, KynA downregulates the expression of P4HA2 to promote the ubiquitination and degradation of HIF-1α, which leads to a decrease in the transcription of HILPDA, and ultimately leads to an increase in lipolysis of colon cancer cells.

**Conclusions:**

Our findings reveal that sleep deprivation impairs intracellular lipolysis by KynA, leading to lipid droplets accumulation in colon cancer cells. This process ultimately promotes colon cancer liver metastasis. This suggests a promising strategy for colon cancer treatment.

## Highlights

• Sleep deprivation promotes colon cancer liver metastasis.
• The gut microbiota metabolite Kynurenic acid is reduced in sleep-deprived mice.
• P4HA2-HIF-1α signal activation and lipid droplet accumulation during sleep deprivation.
• Treatment with Kynurenic acid inhibits colon cancer liver metastasis in sleep-deprived mice.

## Graphical abstract

**Figure:**
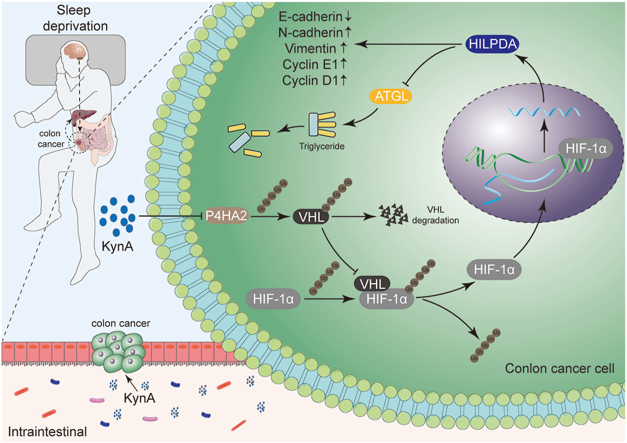

## 1 Introduction

Tumors are often viewed as “organs” regulated by the nervous system [1], and neurological disorders resulting from sleep deprivation are involved in the development and progression of various malignancies [2]. Disturbances in sleep patterns among cancer patients are typically attributed to factors such as cancer-related pain and side effects from treatments [3]. The interplay between sleep disturbances and cancer progression is evident, as epidemiological studies have shown a significantly increased risk of colon cancer among individuals with long-standing sleep disorders (odds ratio = 1.318, 95 % CI 1.121–1.551) [4]. Our previous research has demonstrated that sleep deprivation can promote lung metastasis in colon cancer via the neurotransmitter γ-aminobutyric acid (GABA) [5]. Approximately 50% of colon cancer patients develop liver metastases, which are responsible for the majority of deaths related to the disease [[6], [7], [8]]. However, the specific relationship between sleep deprivation and liver metastases in colon cancer remains unexplored.

The brain-gut-microbiome axis mediates a bidirectional relationship between sleep and gut microbiota [9]. Studies indicate that sleep deprivation can induce gut microbiota dysbiosis, which in turn may influence the progression of central nervous system diseases [10,11] and intestinal diseases [12,13] through its metabolites. Additionally, the microbiome is believed to play a role in the initiation, development, and metastasis of various cancers, affecting treatment response [14,15]. Nonetheless, whether the gut microbiota serves as a link between sleep deprivation and colon cancer needs further exploration.

Kynurenic acid (KynA), a tryptophan metabolite metabolized by the intestinal microbiota and absorbed from the intestine into the bloodstream [16,17], has demonstrated neuroprotective, antiepileptic and potential therapeutic effects on the central nervous system [[18], [19], [20]]. Although extensive research has been conducted on KynA’s role in brain physiology and pathology, peripheral functions remain less understood. Previous studies suggest that KynA may have anti-tumor effects in cancers such as gastric cancer, glioblastoma, colon cancer, and renal cell carcinoma [[21], [22], [23], [24]], but findings are inconsistent. The role of KynA in mediating the relationship between sleep deprivation and colon cancer remains unknown.

Lipids, fundamental components of cells, play critical roles in various cellular activities. Disruption in lipid metabolism is a notable feature of cancer, as cancer cells exploit lipid metabolism to support rapid proliferation, survival, migration, invasion, and metastasis during tumor progression [25]. Evidence suggests that lipid droplet accumulation is a hallmark of hypoxic cancer cells [26], with Hypoxia-inducible lipid droplet associated (HILPDA) protein playing a crucial role in this process by inhibiting adipose triglyceride lipase (ATGL)-mediated intracellular lipolysis [27,28]. However, it remains unclear whether KynA regulates lipid metabolism reprogramming in colon cancer cells through HILPDA.

In this study, we investigated how sleep deprivation affects the gut microbiota metabolite KynA, which is involved in lipid metabolism disorders during liver metastasis of colon cancer, and further revealed its function and potential mechanism of action. Our findings suggest that KynA may be particularly attractive as a therapeutic target for colon cancer as a key factor in the progression of colon cancer promoted by sleep deprivation.

## 2 Methods

### 2.1 Cell culture

The mouse colon cancer cell line (MC38) and the human colon cancer cell lines (LoVo, HCT116) were purchased from American Type Culture Collection (ATCC, USA). All cell lines were cultured with DMEM/high glucose (Gibco, USA). All cell lines were cultured in a medium supplemented with 10 % fetal bovine serum (Gibco, USA) at 37 °C under 5 % CO2 incubator.

### 2.2 Sleep quality survey

We assessed patients' sleep quality using the Pittsburgh Sleep Quality Index (PSQI) Questionnaire [29]. All 25 colorectal cancer patients were randomly selected from the Department of Gastrointestinal Surgery, Union Hospital, Tongji Medical College, Huazhong University of Science and Technology (Wuhan, China). According to the PSQI score, patients were divided into four levels of sleep quality: I. very good (≤5), II. good (6–10), III. poor (11–15), and IV. very poor (≥16). Preoperative imaging results and postoperative pathological results of the patient were collected to evaluate the relationship between patients' sleep and colon cancer progression. The consents were obtained from all patients. The details of patient information are listed in Supplementary Table 1.

### 2.3 Experimental animals

Female C57BL/6J mice (4–6 weeks old) were purchased from Vital River Laboratory Animal Technology Co. Ltd. (Beijing, China). The mice were housed in a specific pathogen-free facility under controlled environmental conditions (23–25 °C, 40 %–60 % humidity). All mice were fed a normal diet. The care and handling of the mice were processed following the National Research Council’s animal care guidelines and approved by the Institution Animal Care and Use Committee of Huazhong University of Science and Technology.

### 2.4 Sleep deprivation mouse model

To establish the sleep deprivation mouse model, we employed a continuously rotating machine, which allows for the artificial adjustment of rotational intensity and duration. Briefly, a disturbance bar located at the bottom of the device rotates and slides at a constant frequency, moving beneath the mouse’s body to prevent sleep. As long as the deprivation time of the sleep deprivation device and the frequency and speed of the disturbance bar rotation are set, the mice can be stably and continuously sleep-deprived. For tests performed on animals maintained under this light cycle, we used the zeitgeber time (ZT) nomenclature, with ZT0 (3:00) set as the light-on time, and ZT20 (23:00) set as the time for light-off. Female C57BL/6J mice (4–6 week old) were randomly divided into an experimental group and a control group. The mice in the experimental group were placed in the sleep deprivation device with a regular 20-hour light (from ZT0 to ZT20)/4-hour dark cycle. In this study, in order to simulate sleep deprivation caused by prolonged cancer pain during cancer treatment, the disturbance bar was set to move every 30 s for 20 h (from ZT0 to ZT20) per day with a working speed of 10 r/min. The mice in the control group had the same diet as the experimental group, except that they were not subject to daily sleep deprivation. This process continued until the end of the experiment.

### 2.5 Subcutaneous xenograft model

The subcutaneous xenograft model of mice was that, after 5 days of sleep deprivation, a total of 1 × 10^6^ colon cancer cells, transfected with overexpression plasmids or not, were suspended in 200 μL PBS and injected into mice subcutaneously. The first grouping arrangement comprises: a Control group (*n* = 5) and an SD group (*n* = 5). The second grouping format entails: a Control + DMSO group (*n* = 5), a Control + KynA group (*n* = 5), an SD + DMSO group (*n* = 5), and an SD + KynA group (*n* = 5). Lastly, the third grouping structure includes: a Control + DMSO + Vector group (*n* = 5), an SD + DMSO + Vector group (*n* = 5), an SD + KynA + Vector group (*n* = 5), and an SD + KynA + P4HA2 group (*n* = 5). Mice in the KynA group were intraperitoneally injected with 100 μL 10 mg/kg KynA every two days. Mice in the DMSO group were intraperitoneally injected with the same dose of DMSO (100 μL, 10 %) as required to solubilize KynA. Mice in the P4HA2 group are injected with MC38 cells transfected with the P4HA2 overexpression plasmid. Mice in the SD group were continuously sleep-deprived until the end of the experiment. After 14 days, all mice were sacrificed. Subcutaneous tumors were peeled, weighed and fixed with 4 % paraformaldehyde for Oil red O staining.

### 2.6 Liver metastasis model

To establish the liver metastasis model of mice, after 5 days of sleep deprivation, we anesthetized the mice with 1 % pentobarbital and then made a small incision to open the left side of the abdomen of mice to expose the spleen. Next, approximately 5 × 10^5^ colon cancer cells, transfected with overexpression plasmids or not, were suspended in 100 μL PBS and injected into the spleen of the mice. The incision was sutured and antibiotics were given to prevent infection. The first grouping arrangement comprises: a Control group (*n* = 3) and an SD group (*n* = 3). The second grouping format entails: a Control + DMSO group (*n* = 3), a Control + KynA group (*n* = 3), an SD + DMSO group (*n* = 3), and an SD + KynA group (*n* = 3). Lastly, the third grouping structure includes: a Control + DMSO + Vector group (*n* = 3), an SD + DMSO + Vector group (*n* = 3), an SD + KynA + Vector group (*n* = 3), and an SD + KynA + P4HA2 group (*n* = 3). Mice in the KynA group were intraperitoneally injected with 100 μL 10 mg/kg KynA every two days. Mice in the DMSO group were intraperitoneally injected with the same dose of DMSO (100 μL, 10 %) as required to solubilize KynA. Mice in the P4HA2 group are injected with MC38 cells transfected with the P4HA2 overexpression plasmid. Mice in the SD group were continuously sleep-deprived until the end of the experiment. All mice were sacrificed after 14 days in order to evaluate liver metastasis. Metastases were fixed with 4% paraformaldehyde for HE staining, Oil red O staining and IHC.

### 2.7 Omics analysis

Untargeted metabolomics analysis: After 19 days of sleep deprivation, the feces of the mice of the control and SD groups were collected. Then, we performed fecal untargeted metabolomics analysis by liquid chromatography-mass spectrometry (LC-MS), gas chromatography-mass spectrometry (GC–MS) and nuclear magnetic resonance (NMR). Total metabolites extraction, metabolites sequencing and bioinformatics data analysis were performed by Novogene Bioinformatics Technology Co., Ltd. (Beijing, China). Sequencing results are listed in Supplementary Table 2.

Transcriptomics analysis: 4 × 10^5^ colon cancer cells (LoVo) were plated in the bottom chamber of 6-well plates and treated by KynA (200 μM) or DMSO for three days. Remove the supernatant, add 1 mL VeZol (Vazyme Biotech Co., Ltd, Nanjing, China), collect all colon cancer cells into a 1.5 mL centrifuge tube and store in a −80 °C freezer for detection as soon as possible. Then, we performed mRNA transcriptome profiling by RNA sequencing. Total RNA extraction, RNA sequencing and bioinformatics data analysis were performed by Beijing Tsingke Biotech Co., Ltd. (Beijing, China). Sequencing results are listed in Supplementary Table 3.

### 2.8 Transfection assay

All the overexpression plasmids targeting HILPDA (pcDNA3.1-HILPDA), HIF-1α (pcDNA3.1-HIF-1α), P4HA2 (pcDNA3.1-P4HA2) and VHL (pcDNA3.1-VHL) were designed and synthesized by GeneChem (Shanghai, China), and the empty plasmid was used as a negative control. All the small interference RNAs targeting HILPDA, HIF-1α, P4HA2 and VHL were designed and synthesized by GeneChem (Shanghai, China).

The small interference RNAs were transfected at a final concentration of 50 nM, and the plasmid was transfected at a final concentration of 1.5 μg for 6 well plates. Lipofectamine 2000 transfection reagent (Thermo Fisher Scientific, USA) was used for cell transfections. First, seed cells to be 70 %–90 % confluent at transfection. Then, dilute Lipofectamine Reagent in Opti-MEM Medium and dilute plasmids or siRNAs in another Opti-MEM Medium. Mix the two and incubate for 5 min at room temperature. Finally, add complex to cells. Protein and total RNA were extracted after 48 h. The sequences of siRNAs are listed in Supplementary Table 4.

### 2.9 Pathological staining

Hematoxylin-eosin staining (HE staining): Mouse liver metastases were embedded in paraffin wax, then cut into 1–10 μm thickness, and paraffin was deparaffined with xylene, anhydrous ethanol, 95 % alcohol, 90 % alcohol, 80 % alcohol, 70 % alcohol and distilled water in turn. Then the cell nucleus and cytoplasm were stained with hematoxylin and eosin successively, and then the plates were dehydrated and sealed for microscopic (Olympus, Japan) examination and image acquisition. Two experienced pathologists evaluated all the results from the HE staining analysis of the tissue sections.

Oil red O staining: Mouse liver metastases and subcutaneous xenografts were embedded in optimal cutting temperature compound. Frozen liver and subcutaneous xenografts sections were cut at 10 μm thickness, fixed with 10 % buffered formalin, dehydrated with 100 % propylene glycol, and stained with 0.5 % Oil Red O (Beyotime, Shanghai, China) for 30 min at 55 °C. Sections were washed repeatedly with 85 % propylene glycol followed by distilled water and stained with hematoxylin. Lipid droplets were stained red. Examined under microscope (Olympus, Japan) and collected images. Two experienced pathologists evaluated all the results from the Oil red O staining analysis of the tissue sections.

Immunohistochemistry (IHC): Mouse liver metastases were formalin-fixed, dehydrated and paraffin-embedded. Then, the tissue sections were incubated with primary antibodies overnight at 4 °C. Next day, the tissue sections were incubated with HRP-conjugated secondary antibodies for 1 h at 37 °C. Then sections were further washed with PBS and distilled water, freshly prepared DAB solution (diaminobenzidine) was subsequently used until the tissue sections were ready to observe. We used the IHC Profiler to evaluate the staining results [30]. The IHC Profiler uses the mean gray value (staining intensity) and the percentage of positive area (stained area) of positive cells as IHC measurements, and finally gives four scores: High positive (4), Positive (3), Low Positive (2) and Negative (1). IHC score was equal to the product of those two. Two experienced pathologists evaluated all the results from the IHC analysis of the tissue sections. The details of primary antibodies are listed in Supplementary Table 5.

### 2.10 Enzyme-linked immunosorbent assay (ELISA)

After mice were subjected to sleep deprivation, blood samples were respectively prepared at ZT1 (4:00), ZT5 (8:00), ZT9 (12:00), ZT13 (16:00), ZT17 (20:00), ZT21 (24:00) and the second day’s ZT1 (4:00) from C57BL/6J mice. Anesthetize the mouse with 1 % pentobarbital, hold the neck of the mouse tightly from the back, congest the retroorbital venous plexus. Then, the blood collector is at an angle of 45 °C with the mouse surface and prick from the inner corner of the eye to collect blood. Finally, immediately centrifuge the blood at 4,000 rpm for 20 min to obtain serum, and store it in a −80 °C freezer for later use. Concentration of KynA in blood were assessed using commercially available ELISA kits (Reed Biotech Ltd, Wuhan, China). Concentration of cortisol and melatonin in blood were assessed using commercially available ELISA kits (Jiangsu Meimian Industrial Co., Ltd, Nanjing, China).

After treatment, proteins that retained enzyme activity were prepared from colon cancer cells. ATGL enzyme activity were assessed using commercially available ELISA kits (Jiangsu Meimian Industrial Co., Ltd, Nanjing, China).

Collected the patient’s blood and immediately centrifuged at 12,000 rpm for 10 min, and took the supernatant and stored it in a −80 °C freezer for later use. The serum KynA concentration were assessed using commercially available ELISA kits (Biorbyt, UK).

Add 50 μL sample and standard to the plate and incubate at 37 °C for 30 min. Wash the plate 5 times with washing solution, add 50 μL enzyme labeling reagent, and incubate at 37 °C for 30 min. Wash the plate 5 times with washing solution, add 50 μL chromogenic solution, and incubate at 37 °C in the dark for 10min. Add 50 μL of stop solution, and use a Quant ELISA Reader to detect the OD value at the wavelength of 450 nm.

### 2.11 Immunofluorescence (IF)

The treated colon cancer cells were placed on a glass slide and fixed with 5 % paraformaldehyde when the cells reached 60 %–70 % confluent. The cells were blocked with 5 % donkey serum for 1 h and then stained with 10 μM Bodipy 493/503 (MedChemExpress, USA) stain in the dark for 40 min. After counterstaining the nuclei with DAPI (Sigma, USA) for 15 min, the fluorescence images were captured by epifluorescence microscopy (Olympus, Japan).

### 2.12 Flow cytometry

LoVo cells and HCT116 cells were inoculated on six-well plates and treated accordingly. The single-cell suspension was prepared when the cell count reached 5 × 10^5^. The cell suspension was fixed with 4 % polyformaldehyde, then the cell suspension was stained with Bodipy 493/503 (MedChemExpress, USA) at the concentration of 10 μM according to the instructions. The intracellular lipid distribution was determined by ID7000™ Spectral Cell Analyzer (Sony, Japan), and the results were analyzed by FlowJo.

### 2.13 Coimmunoprecipitation (co-IP)

After transfection, the cells were lysed in a RIPA buffer (Servicebio, Wuhan, China) at 4 °C for 1 h. The lysate was centrifuged at 15,000×*g* at 4 °C for 15 min and samples were collected from the supernatant as the total lysate for further analysis. To remove non-specific binding, pre-washing with 0.7 μg/μL IgG free BSA and 30–50 μL Protein A/G PLUS agarose. Anti-VHL, Anti-P4HA2 and Anti–HIF–1α were added into the cell lysates respectively and incubated overnight after the addition of Protein A/G PLUS agarose and incubation for 8 h at 4 °C. If necessary, a second round of immunoprecipitation was performed. After washing with 1 mL immunoprecipitation buffer for 5 times, the beads subjected to perform Western blot analysis.

### 2.14 Chromatin immunoprecipitation (ChIP) and DNA gel electrophoresis

ChIP assay was performed using the SimpleChIP® Plus Enzymatic Chromatin IP Kit (CST, USA). Crosslinked the cells with formaldehyde and sonicate to an average size of 300–500 bp. The lysate was added to the EP tube and incubated with the HIF-1α antibody. Purified cross-linked DNA released from protein-DNA complexes and further evaluated eluted DNA by qRT-PCR. Used both input and IgG to confirm that the detected signal comes from a specific binding between chromatin and HIF-1α. Used JASPAR to predict the binding site between HIF-1α and the HILPDA promoter. All ChIP assays were repeated 3 times independently.

After preparing the DNA sample in advance, agarose gel (Thermo Fisher Scientific, USA) was prepared. Put the solidified gel into the electrophoresis tank and prepared for sample loading. Poured the electrophoresis buffer (TAE) (Servicebio, Wuhan, China) into the electrophoresis tank. For loading, added 1 μL loading buffer and 1 μL dye to each well of 5 μL DNA sample, then electrophoresis for 1 h. Removed the gel and then observed it under the ChemiDocTm XRS Molecular Imager System (Bio-Rad, USA).

### 2.15 Cell counting Kit-8 (CCK-8) assay

The CCK-8 was used to assess cell viability in accordance with the manufacturer’s instructions. In brief, cells (2 × 10^3^ per well) were seeded into 96-well plates (200 μL/well) in culture medium supplemented with 10 % FBS with five replicates for each sample. At the appointed time point, 100 μL of fresh medium and 10 μL of CCK-8 solution were added to each well. After incubation for1.5 h at 37 °C, the absorbance was recorded at 450 nm using a Quant ELISA Reader.

### 2.16 Transwell assays

Culture medium (600 μL) containing 10 % FBS was added to the bottom chamber, while 5 × 10^4^ cells were seeded in the top chamber. After 24 h, cells that had passed to the underside of the filter were fixed with methanol and stained with 0.1 % crystal violet, the cells that had no migrated were removed using cotton swabs. Cells were counted under the microscope (Olympus, Japan), and the number of stained cells represented invasiveness.

### 2.17 Colony formation assay

Forty-eight hours after transfection, 500 cells were plated into 6-well plates and cultured for 10 days. Thereafter, the cell colonies were fixed with 4% paraformaldehyde for 20 min and stained with 0.1 % crystal violet for 30 min at room temperature. The cell colonies were then photographed and counted.

### 2.18 Wound healing assays

5 × 10^5^ cells were plated in 6-well plates overnight. 1 mL pipette tips were used to scratch a straight line when the cells achieved 60 %–80 % confluence. Then cells were cultured with medium without FBS. Then, a picture of the cell wound width was taken under the microscope at 0, 24 and 48 h.

### 2.19 RNA isolation and qRT-PCR

Total RNA from cultured cells was extracted using VeZol Reagent #R411 (Vazyme Biotech Co., Ltd, Nanjing, China) reagent for 5 min at room temperature, then centrifuged at 3000×*g* for 15 min at 4 °C to obtain the supernatant. The supernatant was then added to isopropanol and mixed well, then centrifuged at 3000×*g* for 10 min at 4 °C to discard the supernatant. The pellet was washed with absolute ethanol and then added to diethylpyrocarbonate in water to measure RNA concentration (ng/μL). The RNA was then reverse-transcribed by RT Master Mix (Vazyme Biotech Co.,Ltd, Nanjing, China). The qRT-PCR assays were performed using SYBR master mix (Vazyme Biotech Co.,Ltd, Nanjing, China) on the Applied Biosystems StepOne-Plus System (ABI, USA). The expression levels of cellular RNA and mRNA expression were normalized against the housekeeping gene GAPDH. The primer sequences are listed in Supplementary Table 6.

### 2.20 Western blot (WB)

Lysing cells with RIPA buffers (Servicebio, Wuhan, China) containing PMSF (Sigma, USA) and phosphorylase inhibitors (Sigma, USA). First, protein specimens were separated by SDS-PAGE (Beyotime, Shanghai, China). Then, the target protein were transferred to the Nitrocellulose membrane (Millipore, USA). Then, we incubated the Nitrocellulose membrane with the corresponding primary antibody overnight at 4 °C. This was followed by incubation with secondary antibody (CST, USA) for 1-hour the subsequent day. The protein bands were visualized using ECL (Pierce, USA) and collected by the ChemiDocTm XRS Molecular Imager System (Bio-Rad, USA). Finally, the band densities were analyzed by Image J software. The details of primary antibodies are listed in Supplementary Table 5.

### 2.21 Bioinformatics analysis

For analysis of public datasets, RNA-seq-based gene expression data in colon cancer were obtained from the GEO database (GSE10950). For gene expression, *P* < 0.05 was used as the cutoff. Tissue chips (HColA030PG05) were purchased from Shanghai Outdo Biotech Co., Ltd. (Shanghai, China).

### 2.22 Statistical analysis

All data were analyzed by GraphPad Prism 5.0. All quantitative results, including relative expression analysis by qRT-PCR analysis, IHC analysis, cell proliferation assay, cell migration assay were presented as the mean ± standard deviation (SD) of at least three independent experiments render. Means between the two groups were compared using a two-tailed unpaired t-test. *P* < 0.05 was considered to be statistically significant.

## 3 Results

### 3.1 Sleep deprivation promotes colon cancer proliferation and liver metastasis

**Figure 1:**
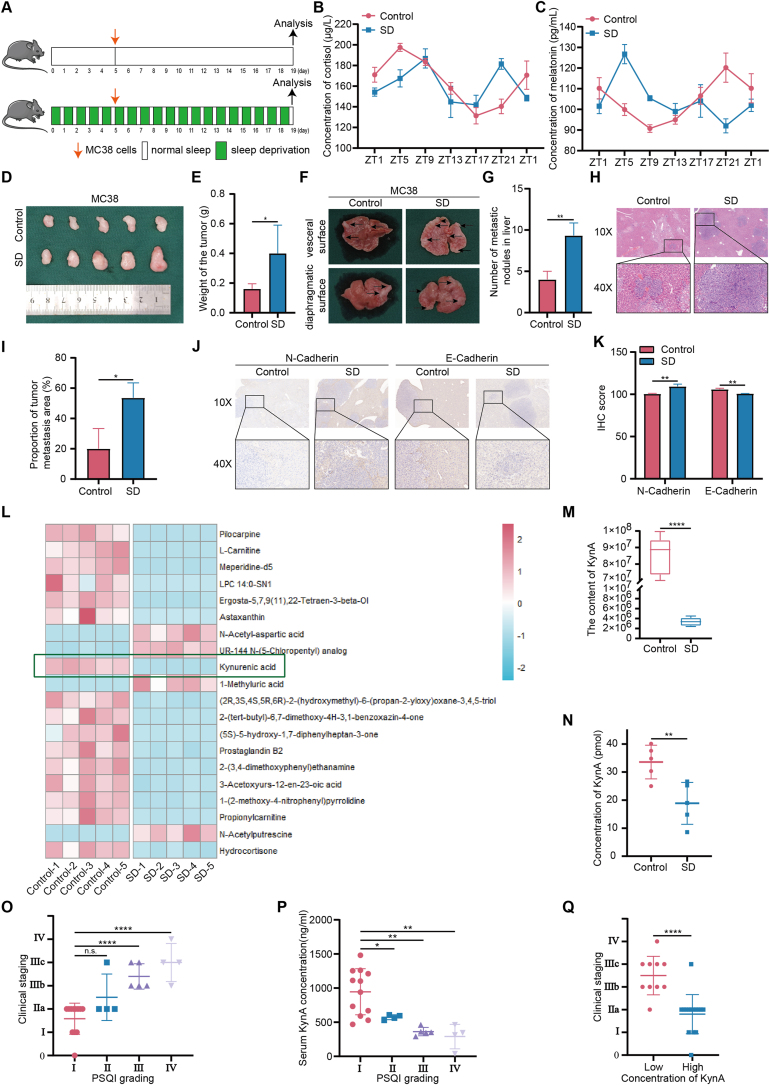
**Sleep deprivation promotes colon cancer proliferation and liver metastasis.A.** Schematic illustration of experimental design of a mouse model of sleep deprivation. **B.** Changes in serum cortisol concentrations over time in mice. **C.** Changes in serum melatonin concentrations over time in mice. **D.** Sleep deprivation increased the size of subcutaneous xenografts. **E.** Subcutaneous xenografts were peeled off and weighed. **F.** Sleep deprivation promoted liver metastases. **G.** The number of liver metastases was counted in the control group and SD group. **H.** HE staining showed liver metastases in mice. **I.** Percentage of infiltrated area of liver metastases. **J.** The expression level of E-cadherin and N-cadherin was detected by IHC in liver metastases of SD group and control group mice. **K.** IHC score of E-cadherin and N-cadherin. **L.** A cluster heatmap of the top 20 metabolites with significant differences in untargeted metabolomics in mouse feces. **M.** Untargeted metabolomics showed the content of KynA in the fecal of SD group and control group mice. **N.** ELISA assay showed the levels of KynA in the serum of SD group and control group mice. **O-Q.** The relationship between sleep status, serum KynA content and tumor progression on colon cancer patients. All data were revealed as mean ± standard deviation for no less than three independent experiments. Significant P values showed as ∗∗∗∗*P* < 0.0001. ∗∗*P* < 0.01. ∗*P* < 0.05. n.s. means the difference was not significant.

We constructed a mouse model of sleep deprivation to explore the effects of sleep deprivation on colon cancer (Figure 1A). We measured the levels of cortisol and melatonin in the serum of mice at different points of the day to validate the success of the sleep deprivation model. Compared with normal mice, the circadian rhythms of cortisol and melatonin were disrupted in sleep-deprived mice (Figure 1B,C). Furthermore, Our results indicated that subcutaneous colon cancer tumors in the sleep-deprived mice were larger and heavier compared to those in the control mice (Figure 1D,E). The average tumor weight in the control group was 0.16 ± 0.04 g, while the average tumor weight in the sleep-deprived group was 0.40 ± 0.19 g. Additionally, the number of liver metastases was significantly greater in the sleep-deprived group (Figure 1F,G). HE staining revealed that the infiltration area of liver metastases was more extensive in the sleep-deprived group compared to the control group (Figure 1H,I). The average infiltration area in the control group was 20.01 ± 13.37 %, while the average infiltration area in the sleep-deprived group was 53.66 ± 9.86 %. Furthermore, the IHC analysis demonstrated that sleep deprivation enhanced metastasis of colon cancer *in situ* (Figure 1J,K).

Our previous studies have shown that sleep deprivation can disrupt gut microbiota and its metabolism [31]. We conducted untargeted metabolomics on mouse fecal samples (Supplementary Table 1) and identified 20 metabolites with significant changes in abundance (Figure 1L), notably finding a significant decrease in KynA levels in the sleep-deprived group (Figure 1M). Additionally, we measured KynA concentrations in the blood of mice and observed a reduction in the sleep-deprived group (Figure 1N).

We further analyzed the relationship between sleep status, serum KynA content and tumor progression on colon cancer patients. The results suggested that the worse the sleep quality, the later the clinical stage of the cancer (Figure 1O), and the lower the serum KynA concentration (Figure 1P). Similarly, patients with lower serum KynA concentrations showed more malignant cancer progression (Figure 1Q).

In conclusion, our findings suggest that sleep deprivation enhances colon cancer proliferation and liver metastasis, with KynA as a potential regulator to be an intermediate mediator within this process.

### 3.2 KynA inhibits *in vivo* metastasis and *in vitro* proliferation and migration of colon cancer

**Figure 2:**
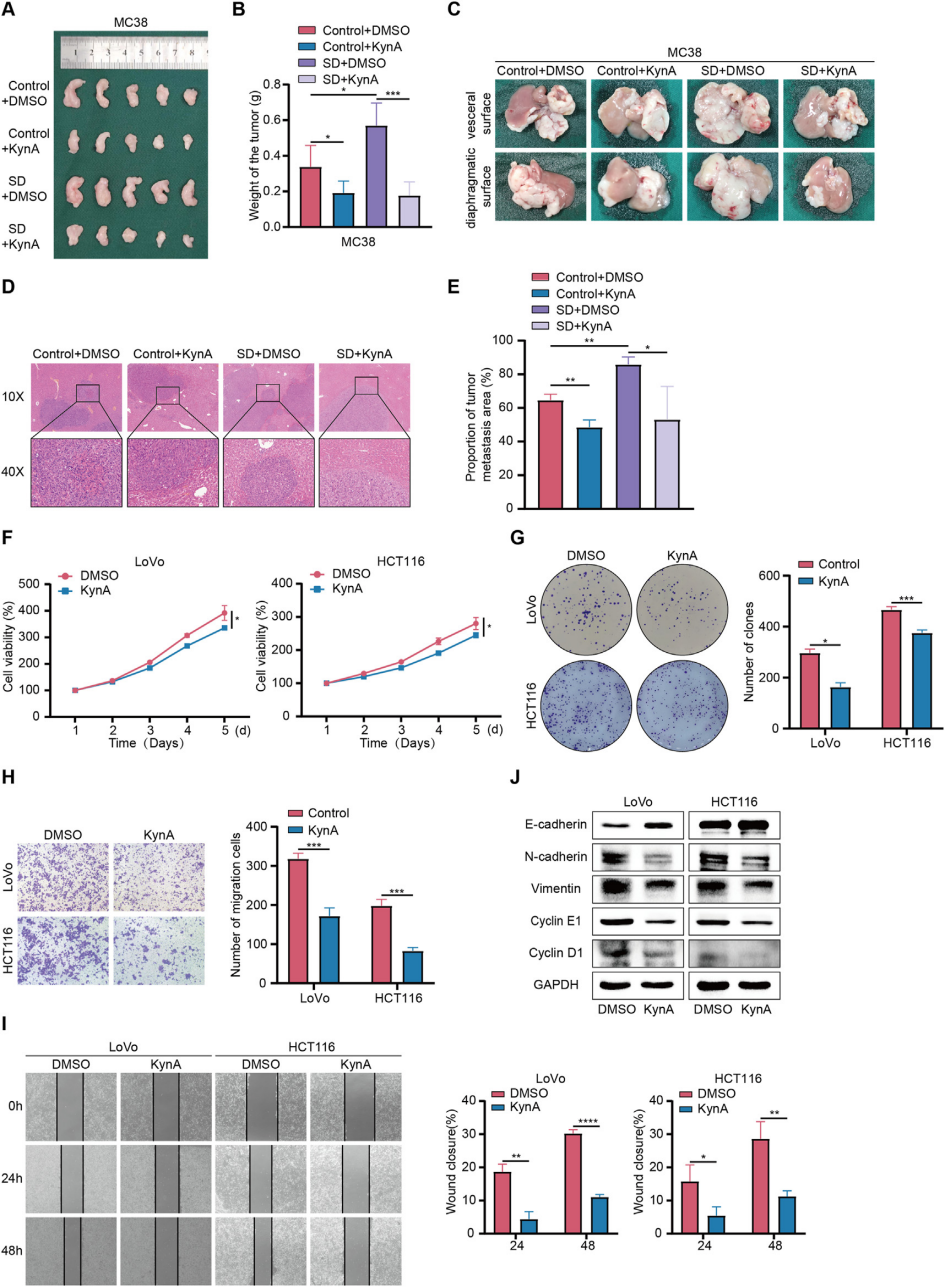
**KynA inhibits *in vivo* metastasis and *in vitro* proliferation and migration of colon cancer.A.** KynA treatment reversed the proliferative effect of sleep deprivation on colon cancer. **B.** Subcutaneous xenografts were peeled off and the weight was measured. **C.** KynA treatment reversed the prometastatic effect of sleep deprivation on colon cancer. **D.** HE staining showed the tumors in the live of mice. **E.** Percentage of infiltrated area of liver metastases. **F.** The proliferation of colon cancer cells was assessed via CCK-8 for 5 days. **G.** The proliferation of colon cancer cells was assessed via colony formation assay for 10 days. **H.** The transwell assays indicated that KynA decreased the migrative ability of colon cancer cells. **I.** The wound healing assays showed that KynA significantly weakened the migrative ability of colon cancer cells. **J.** The expreesion of E-cadherin, N-cadherin, Vimentin, Cyclin E1 and Cyclin D1 in colon cancer cells treated by DMSO or KynA was detected by WB. All data were revealed as mean ± standard deviation for no less than three independent experiments. Significant P values showed as ∗∗∗∗*P* < 0.0001. ∗∗∗*P* < 0.001. ∗∗*P* < 0.01. ∗*P* < 0.05.

**Fig. S1:**
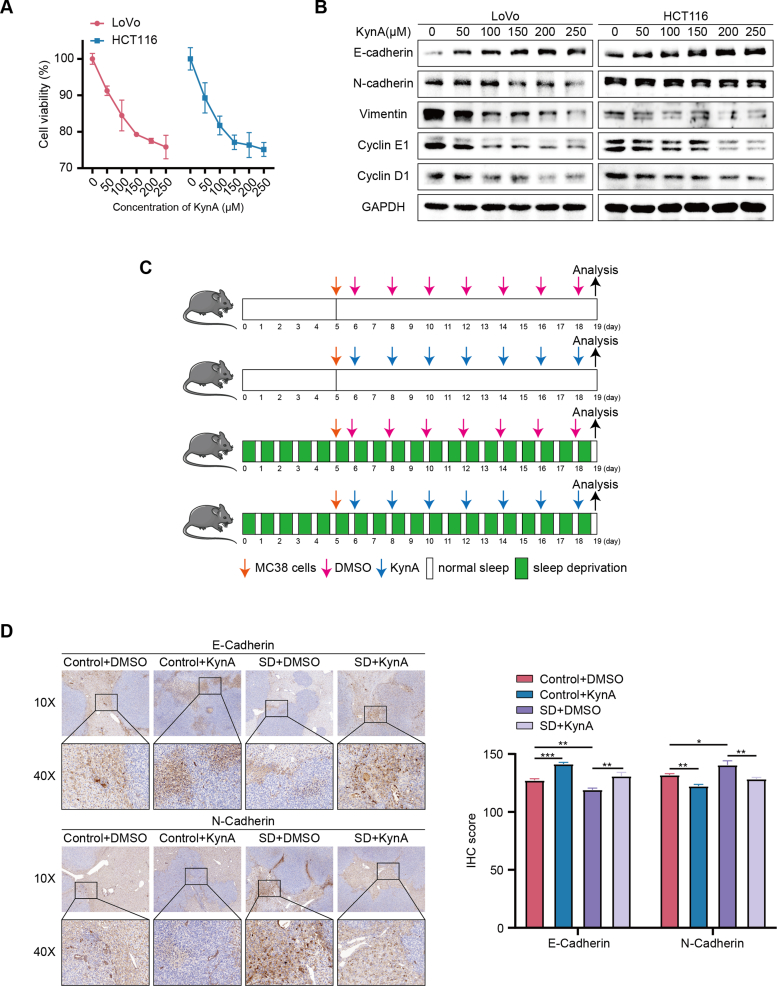
KynA inhibits *in vivo* metastasis and *in vitro* proliferation and migration of colon cancer. **A.** CCK-8 assays showed the cell viability of colon cancer cells treated by different concentration of KynA. **B.** The expression of E-cadherin, N-cadherin, Vimentin, Cyclin E1 and Cyclin D1 in colon cancer cells treated by different concentration of KynA was detected by WB. **C.** Schematic illustration of experiment design of a mouse model of sleep deprivation. DMSO or KynA intraperitoneal injection every two days. **D.** The expression level of E-cadherin and N-cadherin was detected by IHC in liver metastases. All data were revealed as mean ± standard deviation for no less than three independent experiments. Significant P values showed as ∗∗∗*P* < 0.001. ∗∗*P* < 0.01. ∗*P* < 0.05.

To further investigate the impact of KynA on colon cancer progression, we tested various concentrations of KynA on colon cancer cells, ultimately selecting 200 μM KynA for subsequent experiments (Fig. S1A and B). We administered intraperitoneal injection of 100 μL KynA (10 mg/kg) in mice to determine its role as a key regulator in sleep deprivation-induced colon cancer progression (Fig. S1C). The results showed that KynA treatment counteracted the increase in tumor size and weight associated with sleep deprivation (Figure 2A,B). The average tumor weight in Control + DMSO group, Control + KynA group, SD + DMSO group, and SD + KynA group was 0.34 ± 0.12 g, 0.19 ± 0.07 g, 0.57 ± 0.13 g, and 0.18 ± 0.08 g. And KynA treatment reduced the incidence of liver metastases from colon cancer (Figure 2C–E). The average infiltration area in Control + DMSO group, Control + KynA group, SD + DMSO group, and SD + KynA group was 0.65 ± 0.04 %, 0.49 ± 0.04 %, 0.86 ± 0.04 %, and 0.53 ± 0.20 %. Furthermore, the IHC analysis revealed that KynA reversed the enhanced metastasis of colon cancer *in situ* caused by sleep deprivation (Fig. S1D).

In vitro assays, the CCK-8 assays, and colony formation assays demonstrated that KynA inhibited the proliferation ability of colon cancer cells (Figure 2F,G). Additionally, wound healing and Transwell assays showed that KynA reduced the migration ability of these cells (Figure 2H,I). WB analysis indicated that KynA treatment down-regulated the expression of N-cadherin, Vimentin, Cyclin D1, and Cyclin E1 while up-regulating E-cadherin expression (Figure 2J).

In conclusion, our results suggest that KynA inhibits both *in vivo* metastasis and *in vitro* proliferation and migration of colon cancer cells and functions as an intermediate mediator in the process by which sleep deprivation promotes colon cancer progression.

### 3.3 KynA inhibits colon cancer progression by downregulating HILPDA to remodel lipid metabolism

**Figure 3:**
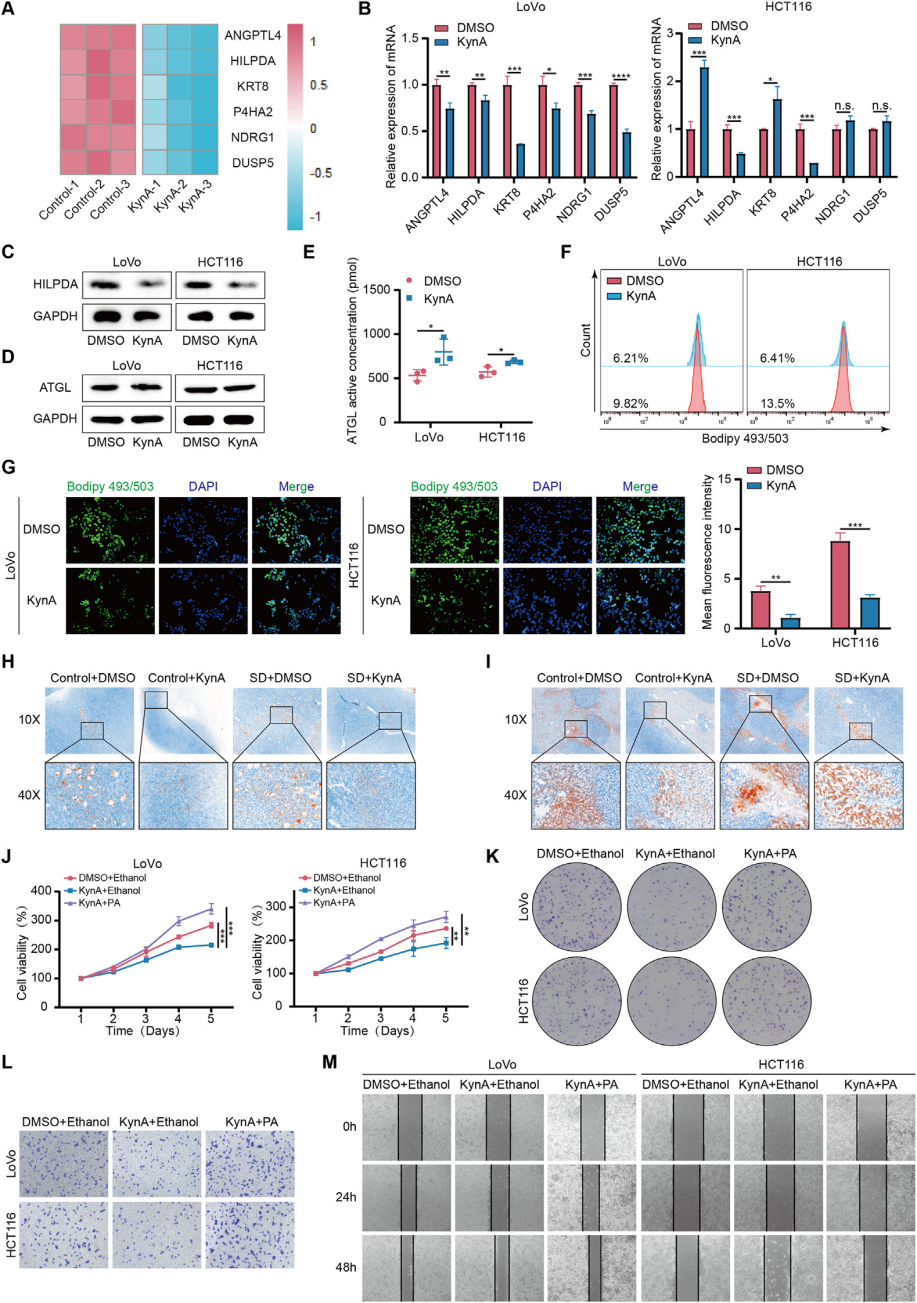
**KynA inhibits colon cancer progression by downregulating HILPDA to remodel lipid metabolism.A.** A cluster heatmap of the expression profiles of mRNAs in DMSO-induced LoVo cells and KynA-induced LoVo cells. **B.** The qRT-PCR analysis verified the expression of ANGPTL4, HILPDA, KRT8, P4HA2, NDRG1 and DUSP5 in DMSO or KynA-treated colon cancer cells. **C.** The expreesion of HILPDA in colon cancer cells treated by DMSO or KynA was detected by WB. **D.** WB results suggested that KynA treatment does not change the expression of ATGL. **E.** ELISA results showed that KynA treatment increased ATGL enzyme activity. **F.** Flow cytometry results suggested that KynA treatment reduces lipid droplets accumulation in colon cancer cells. **G.** IF results suggested that KynA treatment reduces intracellular lipid droplets accumulation in colon cancer cells. **H–I.** The results of oil red O staining showed that sleep deprivation promoted lipid droplets accumulation in subcutaneous xenografts and liver metastases. **J.** CCK-8 assays revealed that PA treatment reversed the KynA-induced inhibition of proliferation in colon cancer cells. **K.** Colony formation assays revealed that PA treatment reversed the KynA-induced inhibition of proliferation in colon cancer cells. **L.** The transwell assays showed that PA treatment reversed the KynA-induced weakened migrative ability of colon cancer cells. **M.** The wound healing assays showed that PA treatment reversed the KynA-induced weakened migrative ability of colon cancer cells. All data were revealed as mean ± standard deviation for no less than three independent experiments. Significant P values showed as ∗∗∗∗*P* < 0.0001. ∗∗∗*P* < 0.001. ∗∗*P* < 0.01. ∗*P* < 0.05. n.s. means the difference was not significant.

**Fig. S2:**
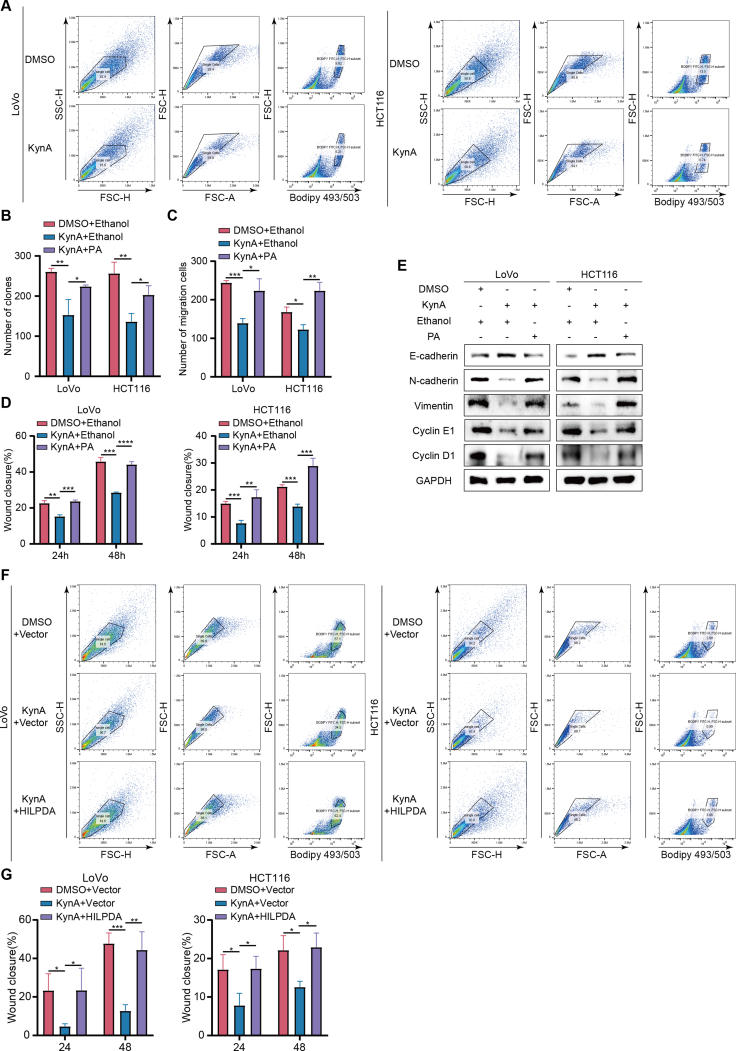
KynA inhibits colon cancer progression by downregulating HILPDA to remodel lipid metabolism. **A.** Identification of Bodipy 493/503+ colon cancer cells. **B.** Colony formation assays revealed that PA treatment reversed the KynA-induced inhibition of proliferation in colon cancer cells. **C.** The transwell assays showed that PA treatment reversed the KynA-induced weakened migrative ability of colon cancer cells. **D.** The wound healing assays showed that PA treatment reversed the KynA-induced weakened migrative ability of colon cancer cells. **E.** The results of WB indicated that PA treatment reversed the KynA-induced inhibition of proliferation and migration in colon cancer cells. **F.** Identification of Bodipy 493/503+ colon cancer cells. **G.** Wound healing assays revealed that overexpression of HILPDA reversed the KynA-induced inhibition of migration in colon cancer cells. All data were revealed as mean ± standard deviation for no less than three independent experiments. Significant P values showed as ∗∗∗∗*P* < 0.0001. ∗∗∗*P* < 0.001. ∗∗*P* < 0.01. ∗*P* < 0.05.

To further explore the molecular mechanism, we performed large-scale mRNA sequencing in colon cancer cells (Supplementary Table 2). This analysis identified 6 mRNAs with significantly altered expression (Figure 3A). These findings were validated using qRT-PCR (Figure 3B). We further examined the effect of KynA on HILPDA expression using WB (Figure 3C). Previous research has shown that HILPDA acts as an inhibitor of ATGL [32]. Similarly, our results indicated that KynA decreases intracellular lipid droplet accumulation by enhancing ATGL enzyme activity rather than altering its expression (Figure 3D–G, Fig. S2A). Additionally, oil red O staining of subcutaneous xenografts and liver metastases showed that sleep deprivation increased lipid droplet accumulation in colon cancers, which KynA effectively reverses (Figure 3H,I). Treatment with Palmitoleic acid (PA), one of the most abundant fatty acids in serum and tissues, was found to reverse KynA’s inhibitory effect on colon cancer cells (Figure 3J-M, Fig. S2B-E). These results suggest that KynA inhibits colon cancer progression by reducing lipid droplet accumulation in colon cancer cells.

**Figure 4:**
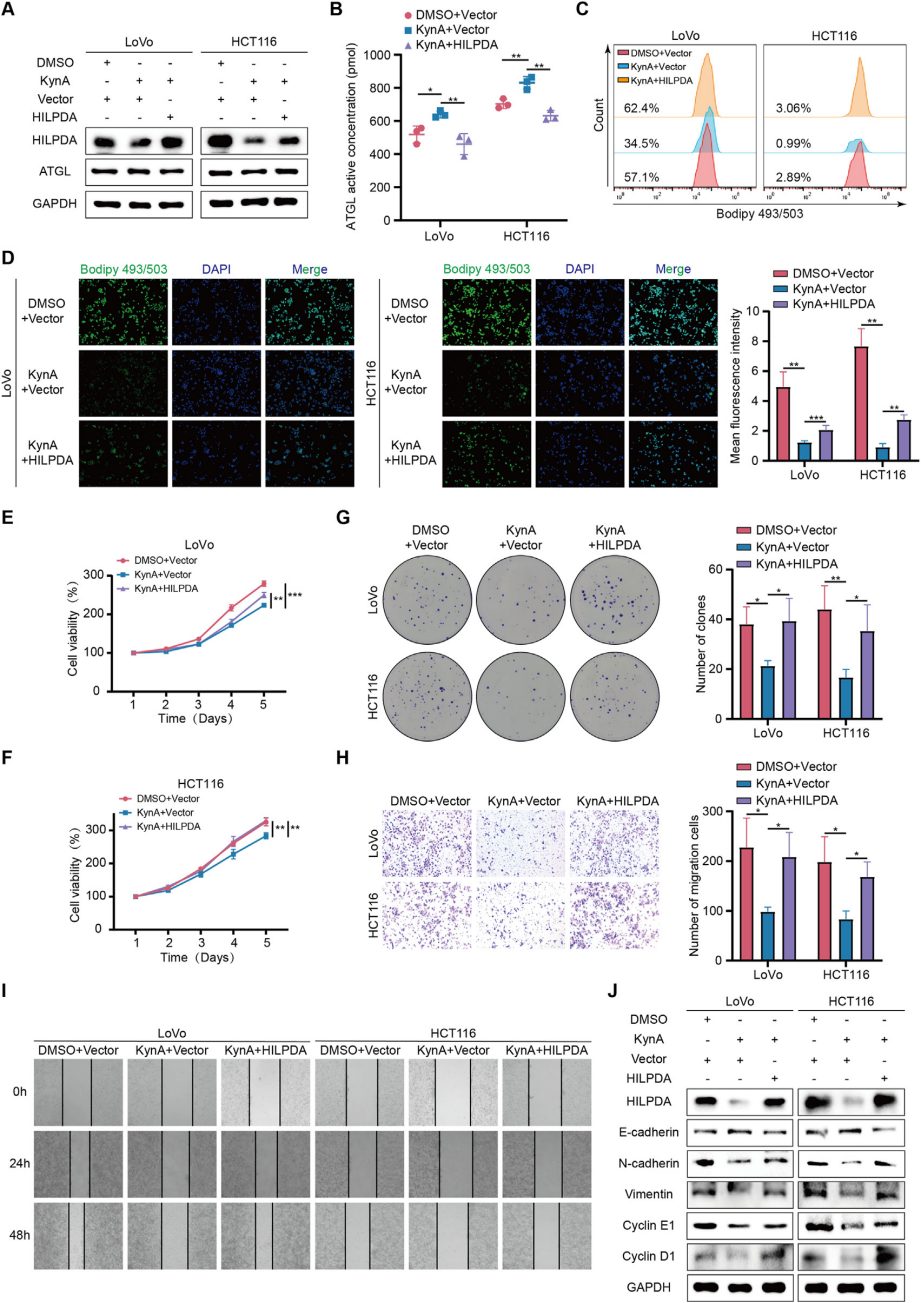
**KynA inhibits colon cancer progression by downregulating HILPDA to remodel lipid metabolism.A.** The results of WB showed that overexpression of HILPDA or not did not change the expression of ATGL. **B.** ELISA assays indicated that overexpression of HILPDA reversed the KynA-induced increase of ATGL enzyme activity in colon cancer cells. **C-D.** Flow cytometry and IF results showed that overexpression of HILPDA reversed the KynA-induced reduction of lipid droplets accumulation in colon cancer cells. **E-G.** CCK-8 assays and colony formation assays revealed that overexpression of HILPDA reversed the KynA-induced inhibition of proliferation in colon cancer cells. **H–I.** The transwell assays and wound healing assays showed that overexpression of HILPDA reversed the KynA-induced weakened migrative ability of colon cancer cells. **J.** The results of WB indicated that overexpression of HILPDA reversed the KynA-induced inhibition of proliferation and migration in colon cancer cells. All data were revealed as mean ± standard deviation for no less than three independent experiments. Significant P values showed as ∗∗∗*P* < 0.001. ∗∗*P* < 0.01. ∗*P* < 0.05.

To determine if HILPDA is a key molecule in KynA-mediated lipid metabolism reprogramming, we constructed a HILPDA gene overexpression plasmid and transfected it into colon cancer cells. Results indicated that HILPDA overexpression counteracted the increase in ATGL activity induced by KynA, leading to increased lipid accumulation in colon cancer cells (Figure 4A–D, Fig. S2F). Additionally, CCK-8 assays, colony formation assays, wound healing assays, Transwell assays, and WB demonstrated that HILPDA overexpression reversed KynA’s inhibitory effects on colon cancer cells (Figure 4E–J, Fig. S2G).

In conclusion, our results suggest that KynA inhibits colon cancer progression by downregulating HILPDA to remodel lipid metabolism.

### 3.4 HIF-1α is involved in KynA-induced lipid metabolism reprogramming by regulating HILPDA transcription

**Figure 5:**
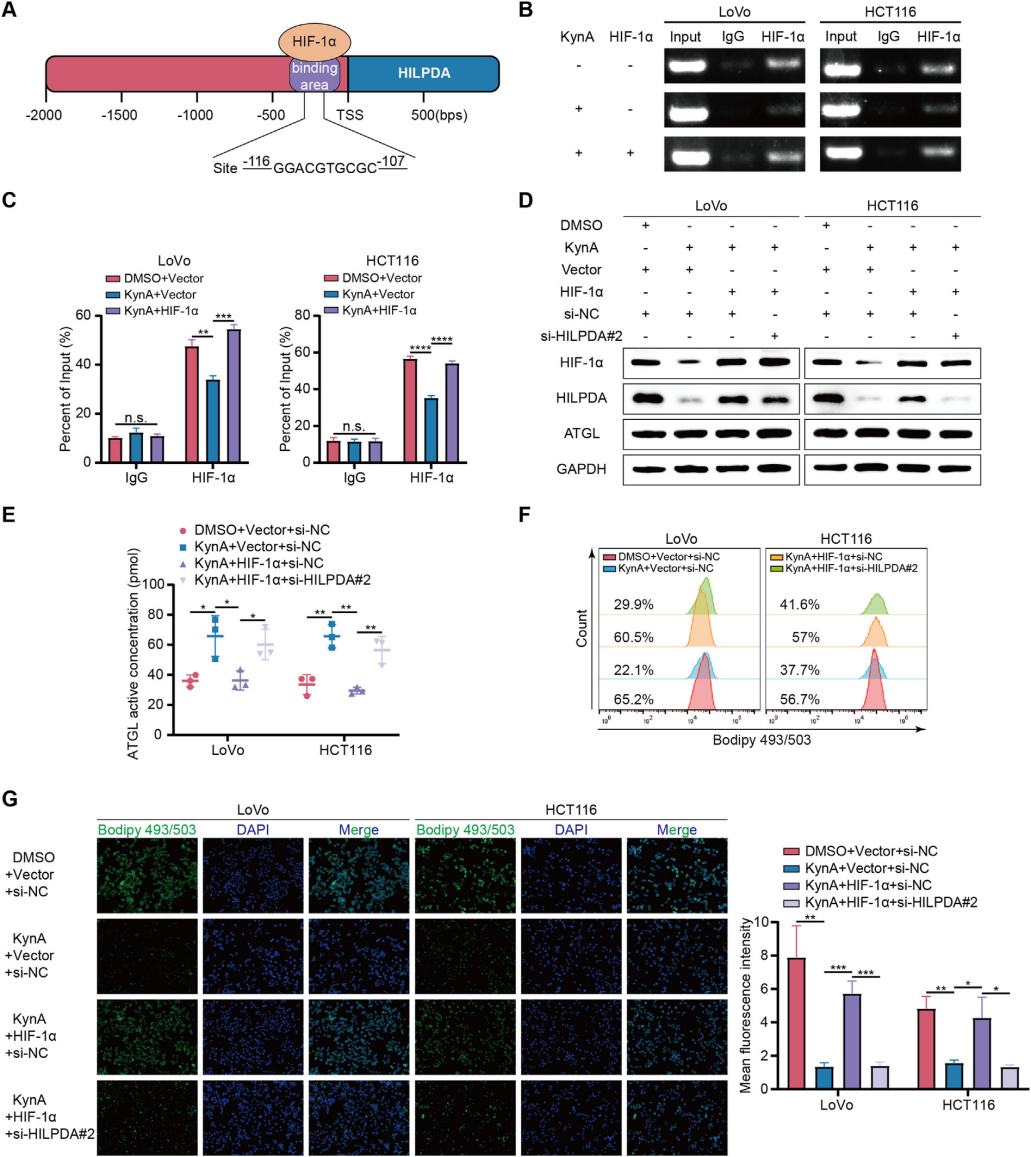
**HIF-1α is involved in KynA-induced lipid metabolism reprogramming by regulating HILPDA transcription.A.** The schematic diagram exhibited one predicted binding site between HIF-1α and the HILPDA promoter. **B–C.** ChIP assays with HIF-1α antibody or IgG were performed to verify binding between HIF-1α and the HILPDA promoter and indicated that overexpression of HIF-1α reversed the KynA-induced downregulation of HILPDA in colon cancer cells. **D.** The expression of HIF-1α, HILPDA and ATGL in colon cancer cells transfected with HIF-1α overexpression plasmid and si-HILPDA#2 or not was detected by WB. **E.** ELISA assays showed that overexpression of HIF-1α reversed the effect of KynA on the increase of ATGL enzyme activity in colon cancer cells, while overexpression of HIF-1α lost its reversal effect when HILPDA was knocked down. **F-G.** Flow cytometry and IF results showed that overexpression of HIF-1α reversed the effect of KynA on the reduction of lipid droplets accumulation in colon cancer cells, while overexpression of HIF-1α lost its reversal effect when HILPDA was knocked down. All data were revealed as mean ± standard deviation for no less than three independent experiments. Significant P values showed as ∗∗∗∗*P* < 0.0001. ∗∗∗*P* < 0.001. ∗∗*P* < 0.01. ∗*P* < 0.05. n.s. means the difference was not significant.

Previous studies have shown that HILPDA is regulated by HIF-1α [27] and that HIF-1α functions as a transcription factor in tumor progression [33]. Using the JASPAR database (https://jaspar.elixir.no/), we predicted potential transcription factors for HILPDA, with HIF-1α among the top candidates. We further used a bioinformatics tool (https://www.ncbi.nlm.nih.gov/) to identify binding sites for HIF-1α and HILPDA (Figure 5A). ChIP assays and DNA gel electrophoresis revealed that the HIF-1α–bound complex significantly enriched the HILPDA promoter compared to the IgG control. Additionally, overexpression of HIF-1α reversed the KynA-induced reduction in HILPDA promoter enrichment (Figure 5B,C). WB confirmed that overexpression of HIF-1α counteracted the KynA-induced reduction in HILPDA expression (Figure 5D).

**Fig. S3:**
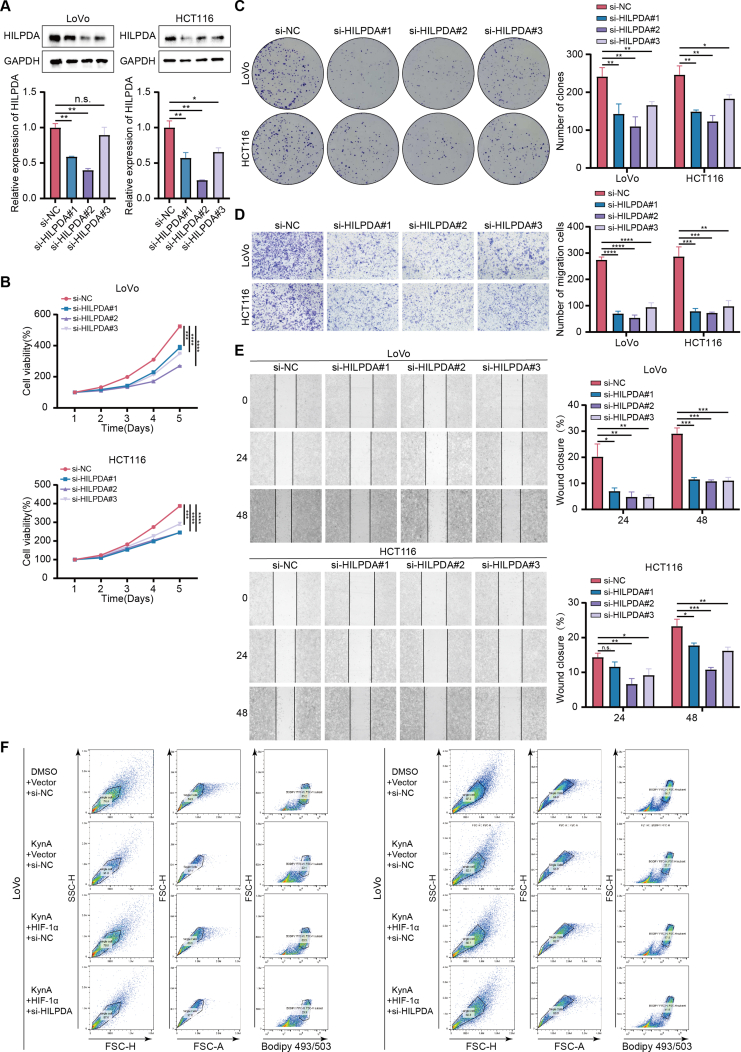
HIF-1α is involved in KynA-induced lipid metabolism reprogramming by regulating HILPDA transcription. **A.** WB and qRT-PCR were used to verify the knockdown ability of siRNAs of HILPDA. **B–C.** CCK-8 assays and colony formation assays revealed that knockdown of HILPDA inhibited proliferation of colon cancer cells. **D-E.** The transwell assays and wound healing assays showed that knockdown of HILPDA weakened migrative ability of colon cancer cells. **F.** Identification of Bodipy 493/503+ colon cancer cells. All data were revealed as mean ± standard deviation for no less than three independent experiments. Significant P values showed as ∗∗∗∗*P* < 0.0001. ∗∗∗*P* < 0.001. ∗∗*P* < 0.01. ∗*P* < 0.05. n.s. means the difference was not significant.

To further investigate whether HIF-1α is involved in KynA-HILPDA-mediated lipid metabolism reprogramming, we constructed three siRNAs to knock down HILPDA expression, verifying knockdown efficiency by qRT-PCR and WB (Fig. S3A). Then, the results of CCK-8 assays and colony formation assays showed that knockdown of HILPDA inhibited the proliferation of colon cancer cells (Fig. S3B and C). The results of Transwell assays and wound healing assays showed that knockdown of HILPDA inhibited the migration of colon cancer cells (Fig. S3D and E). WB and ELISA results indicated that overexpression of HIF-1α could reverse the increase in ATGL enzyme activity caused by KynA. However, this reversal effect was lost when HILPDA was knocked down (Figure 5E). The expression level of ATGL remained unchanged across all conditions (Figure 5D). Additionally, IF and flow cytometry results showed that overexpression of HIF-1α could counteract the effects of KynA, leading to increased lipid droplets accumulation in tumor cells. This effect was diminished when HILPDA was knocked down (Figure 5F,G, Fig. S3F).

In conclusion, our findings suggest that HIF-1α regulates HILPDA transcription and is involved in the reprogramming of lipid metabolism.

### 3.5 KynA promotes the degradation of HIF-1α via the ubiquitin-proteasome pathway by targeting P4HA2

**Figure 6:**
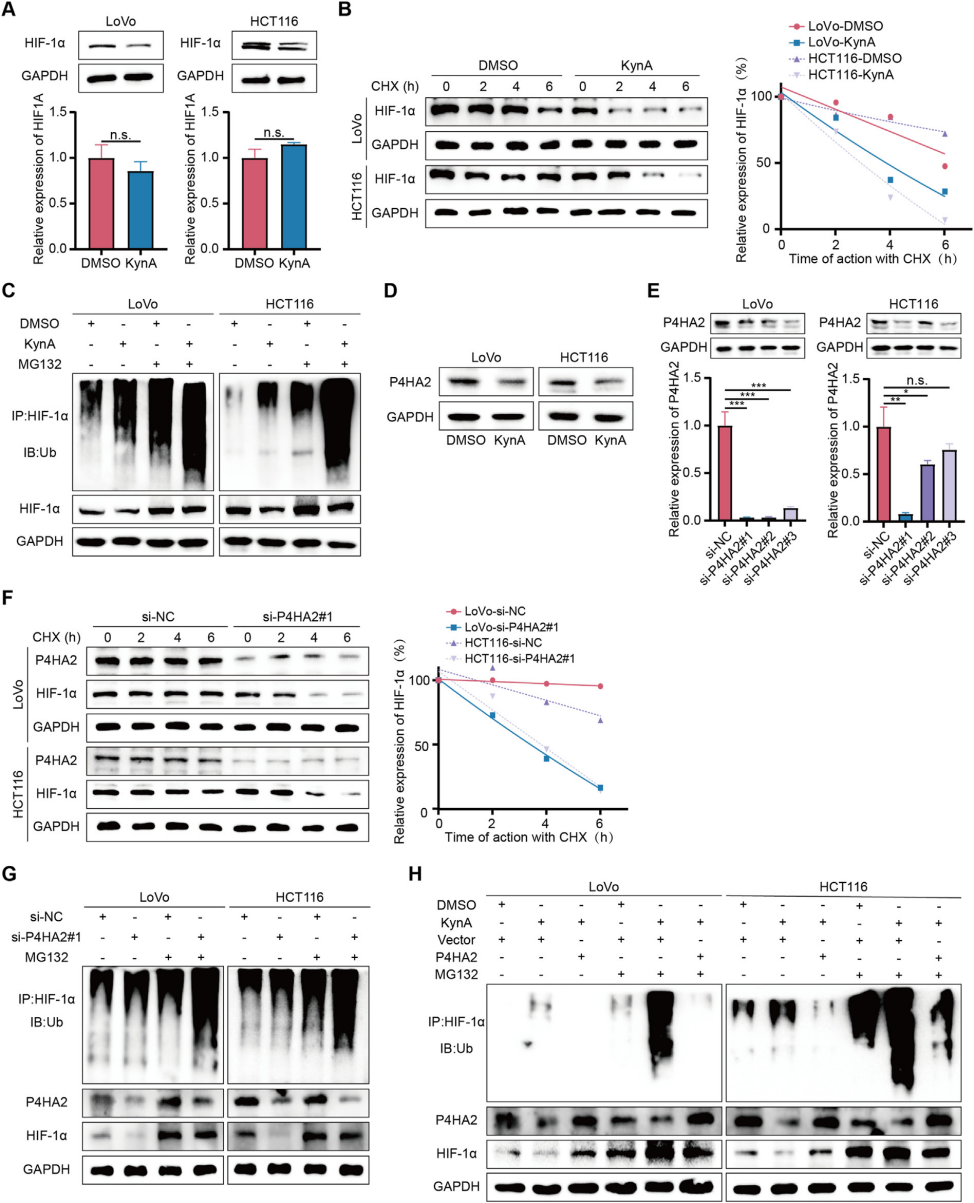
**KynA promotes the degradation of HIF-1α via the ubiquitin-proteasome pathway by targeting P4HA2.A.** The expreesion of HIF-1α in colon cancer cells treated by DMSO or KynA was detected by WB and qRT-PCR. **B.** Colon cancer cells were treated with KynA followed by treatment with CHX for the indicated times. The intensity of HIF-1α expression at each time point was quantified by densitometry and plotted against time. **C.** Ubiqutin assays of colon cancer cells treated with KynA for 3 days and then incubated with or without MG132 for 6 h. **D.** The results of WB verified that KynA treatment down-regulated the expression of P4HA2. **E.** WB and qRT-PCR were used to verify the knockdown ability of siRNAs of P4HA2. **F.** Colon cancer cells were transfected with si-P4HA2#1 or si-NC followed by treatment with CHX for the indicated times. The intensity of HIF-1α expression at each time point was quantified by densitometry and plotted against time. **G.** Ubiqutin assays of colon cancer cells transfected with si-P4HA2#1 or si-NC and then incubated with or without MG132. **H.** Ubiqutin assays indicated that overexpression of P4HA2 in the presence of MG132 reversed the effect of KynA in promoting the ubiquitination of HIF-1α. All data were revealed as mean ± standard deviation for no less than three independent experiments. Significant P values showed as ∗∗∗*P* < 0.001. ∗∗*P* < 0.01. ∗*P* < 0.05. n.s. means the difference was not significant.

Our experiments revealed that KynA treatment downregulates HIF-1α expression levels of protein in colon cancer cells without affecting HIF-1α mRNA expression, indicating a post-transcriptional regulation of HIF-1α (Figure 6A). To determine if KynA affects HIF-1α ubiquitination, we treated colon cancer cells with a protein synthesis inhibitor cycloheximide (CHX). After CHX treatment, the degradation rate of HIF-1α protein was significantly higher in the KynA treatment group compared to the DMSO treatment group (Figure 6B). Furthermore, treatment with MG132, a proteasome inhibitor, followed by immunoprecipitated and detection of ubiquitinated HIF-1α, revealed that KynA increased the ubiquitinated form of HIF-1α in the presence of MG132 compared to the DMSO control (Figure 6C). These results indicate that KynA enhances the degradation of HIF-1α through the ubiquitin-proteasome pathway.

To further understand the specific mechanism by which KynA regulated HIF-1α ubiquitination, we considered P4HA2, a known regulator of HIF-1α protein stability [34]. Our mRNA sequencing results identified P4HA2 as significantly different between both experimental groups (Figure 3A,B). WB results showed that KynA treatment reduced P4HA2 expression (Figure 6D). We then constructed three siRNAs to interfere with the P4HA2 gene expression and verified knockdown efficiency by qRT-PCR and WB (Figure 6E), selecting si-P4HA2#1 for further experiments. Transfection of colon cancer cells with si-P4HA2#1 and CHX treatment revealed that HIF-1α stability decreased in the P4HA2 knockdown group (Figure 6F). Additionally, P4HA2 knockdown significantly increased the ubiquitinated form of endogenous HIF-1α in the presence of MG132 (Figure 6G), while P4HA2 overexpression reversed KynA’s effect on HIF-1α ubiquitination and reduced its ubiquitinated forms (Figure 6H). These results suggest that P4HA2 mediates the ubiquitination of HIF-1α.

In conclusion, our results suggest that KynA promotes the degradation of HIF-1α via the ubiquitin-proteasome pathway by targeting P4HA2.

### 3.6 P4HA2 mediates KynA-induced HIF-1α ubiquitination by regulating VHL ubiquitination

**Figure 7:**
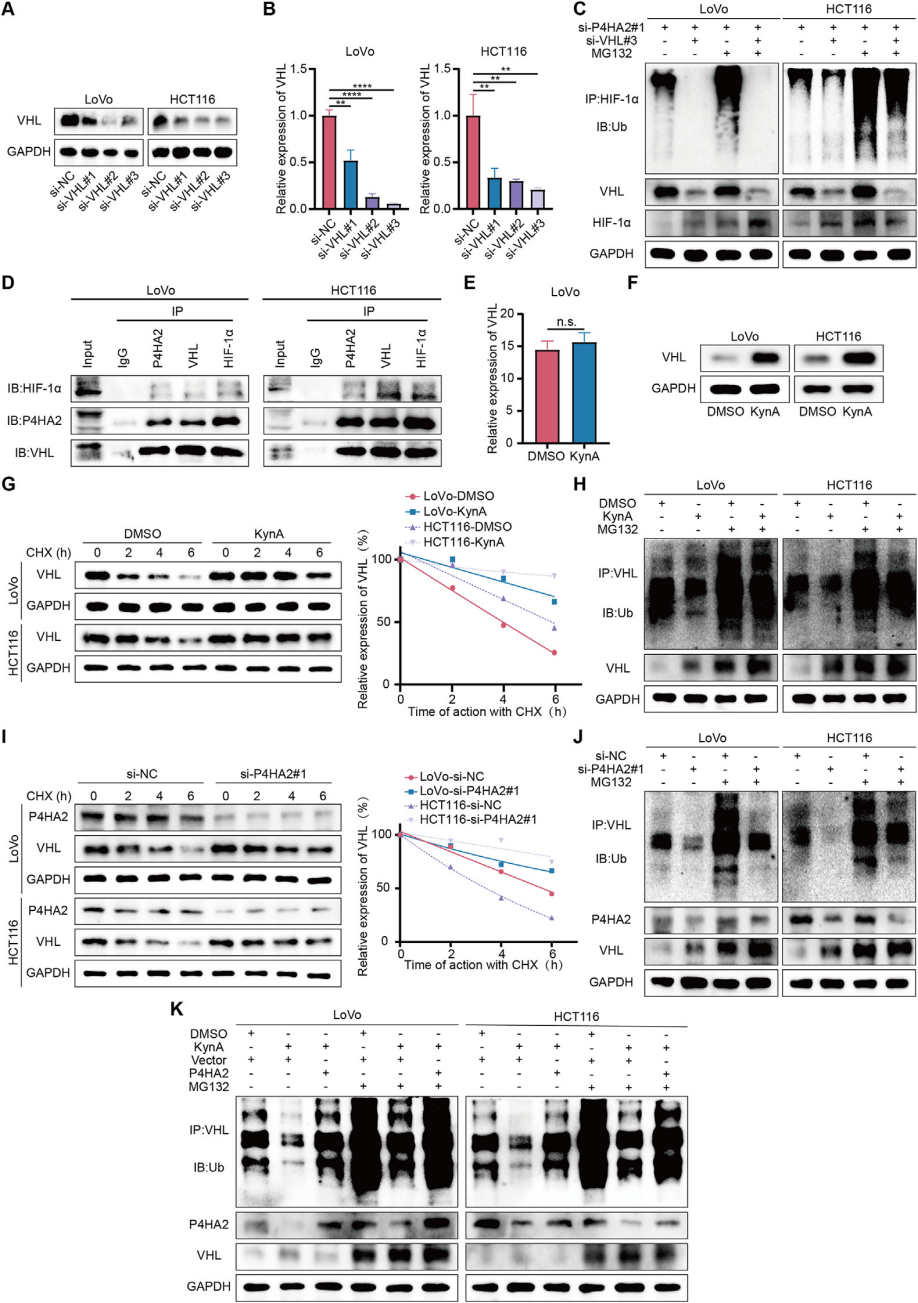
**P4HA2 mediates KynA-induced HIF-1α ubiquitination by regulating VHL ubiquitination.A-B.** WB and qRT-PCR were used to verify the knockdown ability of siRNAs of VHL. **C.** Ubiqutin assays indicated that knockdown of P4HA2 in the presence of MG132 loses its effect in promoting HIF-1α ubiquitination when VHL is knocked down. **D.** Co-IP and WB showed that endogenous P4HA2, VHL and HIF-1α bind to each other. **E.** The results of mRNA sequencing showed that KynA treatment did not change the expression of VHL at RNA level. **F.** The results of WB verified that KynA treatment up-regulated the expression of VHL at protein level. **G.** Colon cancer cells were treated with KynA followed by treatment with CHX for the indicated times. The intensity of VHL expression at each time point was quantified by densitometry and plotted against time. **H.** Ubiqutin assays of colon cancer cells treated with KynA or DMSO and then incubated with or without MG132. **I.** Colon cancer cells were transfected with si-P4HA2#1 or si-NC followed by treatment with CHX for the indicated times. The intensity of VHL expression at each time point was quantified by densitometry and plotted against time. **J.** Ubiqutin assays of colon cancer cells transfected with si-P4HA2#1 or si-NC and then incubated with or without MG132. **K.** Ubiqutin assays indicated that overexpression of P4HA2 in the presence of MG132 reversed the effect of KynA in inhibition the ubiquitination of VHL. All data were revealed as mean ± standard deviation for no less than three independent experiments. Significant P values showed as ∗∗∗∗*P* < 0.0001. ∗∗*P* < 0.01. n.s. means the difference was not significant.

E3 ligases are crucial for the ubiquitination process, and VHL is a well-known E3 ligase that regulates the degradation of HIF-1α [35]. We investigated whether P4HA2 regulates HIF-1α ubiquitination by targeting the E3 ligase VHL. We constructed three siRNAs targeting VHL gene expression and confirmed their knockdown efficiency by qRT-PCR and WB (Figure 7A,B), selecting si-VHL#3 for further experiments. The results showed that VHL knockdown in the presence of MG132 significantly reduced the ubiquitinated form of endogenous HIF-1α (Figure 7C), indicating that VHL mediates P4HA2’s regulation of HIF-1α ubiquitination.

To further understand the relationship between P4HA2, VHL, and HIF-1α, we conducted co-IP assays. Notably, these three proteins were found to interact with each other (Figure 7D). We then examined the regulatory relationship between P4HA2 and VHL. While mRNA sequencing indicated that KynA treatment did not affect the VHL expression at the RNA level (Figure 7E), WB results showed that KynA treatment increased VHL protein levels (Figure 7F), suggesting that VHL is regulated at the post-transcriptional level. Previous studies have shown that E3 ligase can undergo autoubiquitination [36]. We explored whether KynA can influence the autoubiquitination of VHL. After CHX treatment, we observed that VHL protein degradation was slower in the KynA-treated group (Figure 7G). Furthermore, KynA treatment reduced the ubiquitinated form of endogenous VHL protein in the presence of MG132 (Figure 7H). Additionally, P4HA2 knockdown increased VHL protein stability (Figure 7I) and decreased the ubiquitinated form of endogenous VHL protein (Figure 7J), while P4HA2 overexpression reversed KynA’s inhibition of VHL ubiquitination (Figure 7K).

In conclusion, our results suggest that P4HA2 mediates KynA-induced HIF-1α ubiquitination by regulating VHL ubiquitination.

### 3.7 Sleep deprivation promotes colon cancer proliferation and liver metastasis via KynA/P4HA2/VHL/HIF-1α/HILPDA axis

**Fig. S4:**
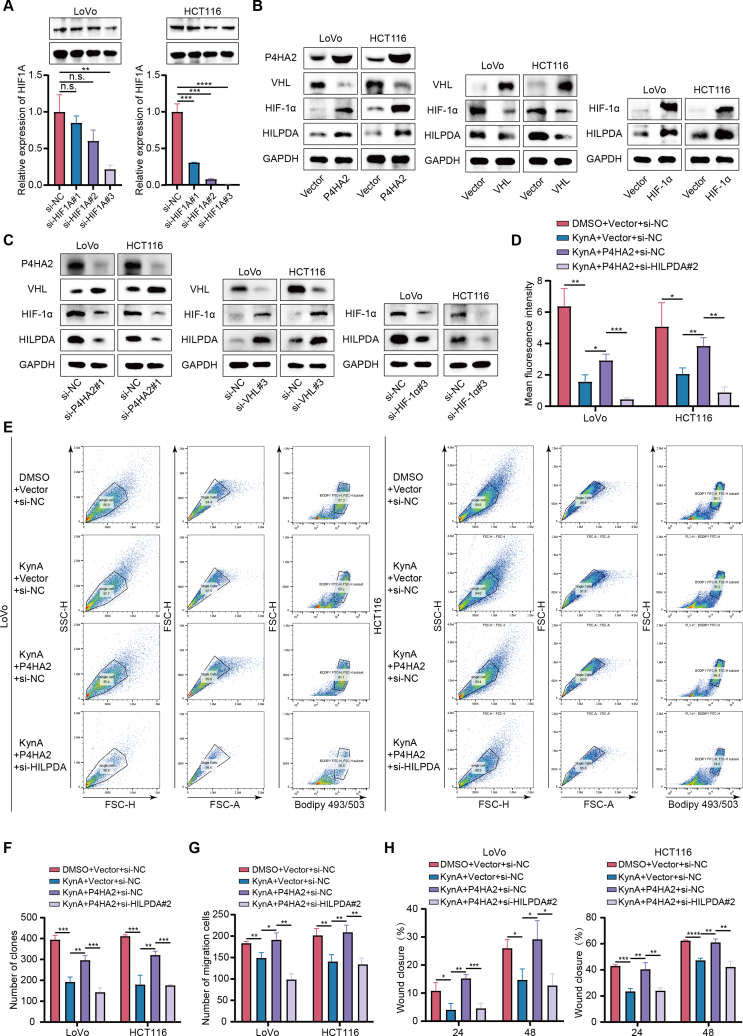
Sleep deprivation promotes colon cancer proliferation and liver metastasis via KynA/P4HA2/VHL/HIF-1α/HILPDA axis. **A.** WB and qRT-PCR were used to verify the knockdown ability of siRNAs of HIF-1α. **B–C.** The expression of P4HA2, VHL, HIF-1α and HILPDA in colon cancer cells transfected with siRNAs or overexpression plasmids was detected by WB. **D.** IF results showed that overexpression of P4HA2 reversed the effect of KynA on the reduction of lipid accumulation in colon cancer cells, while overexpression of P4HA2 lost its reversal effect when HILPDA was knocked down. **E.** Identification of Bodipy 493/503+ colon cancer cells. **F.** Colony formation assays revealed that overexpression of P4HA2 reversed the KynA-induced inhibition of proliferation in colon cancer cells, while overexpression of P4HA2 lost its reversal effect when HILPDA was knocked down. **G-H.** The transwell assays and wound healing assays showed that overexpression of P4HA2 reversed the KynA-induced weakened migrative ability of colon cancer cells, while overexpression of P4HA2 lost its reversal effect when HILPDA was knocked down. All data were revealed as mean ± standard deviation for no less than three independent experiments. Significant P values showed as ∗∗∗∗*P* < 0.0001. ∗∗∗*P* < 0.001. ∗∗*P* < 0.01. ∗*P* < 0.05. n.s. means the difference was not significant.

To investigate the presence of KynA/P4HA2/VHL/HIF-1α/HILPDA axis in mediating the effects of sleep deprivation on colon cancer, we underwent rescue experiments. We constructed three siRNAs targeting the HIF-1α gene and verified their knockdown efficiency by qRT-PCR and WB (Fig. S4A), selecting si–HIF–1α#3 for subsequent experiments. Colon cancer cells were transfected with siRNAs and overexpression plasmids to investigate their mutual regulatory relationships. Our results confirmed similar results to our prior experiments, with P4HA2 being positively correlated with HIF-1α and HILPDA while negatively correlated with VHL (Fig. S4B and C).

**Figure 8:**
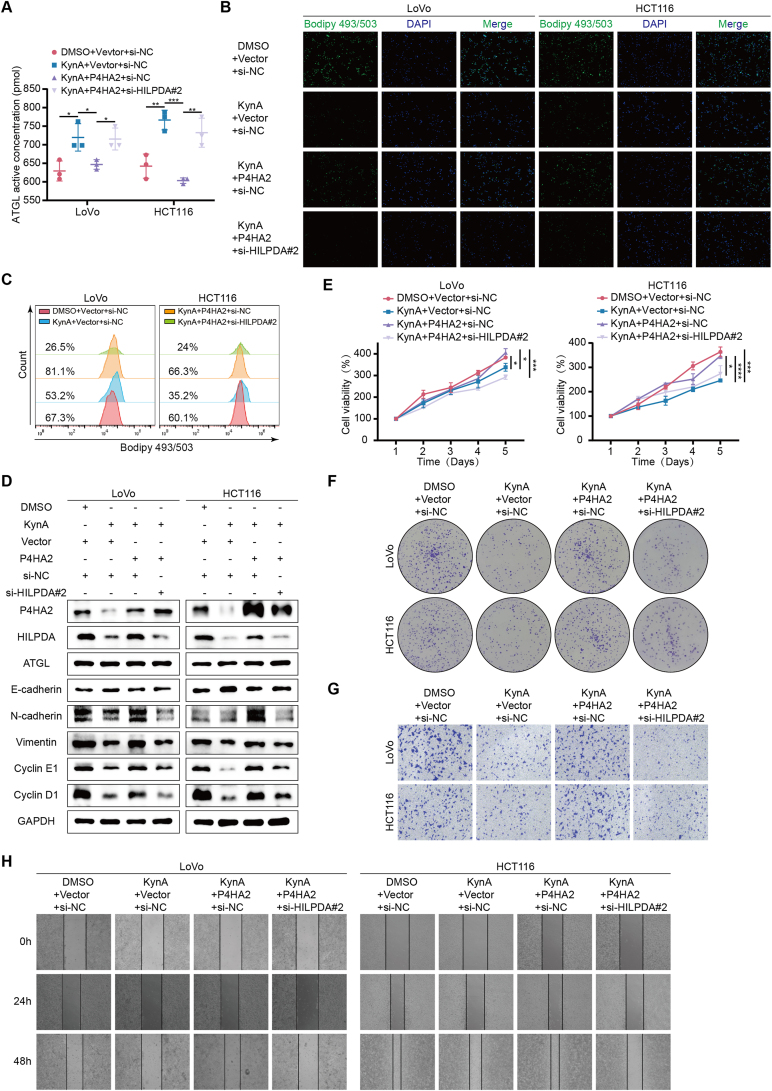
**Sleep deprivation promotes colon cancer proliferation and liver metastasis via KynA/P4HA2/VHL/HIF-1α/HILPDA axis.A.** ELISA assays showed that overexpression of P4HA2 reversed the effect of KynA on the increase of ATGL enzyme activity in colon cancer cells, while overexpression of P4HA2 lost its reversal effect when HILPDA was knocked down. **B–C.** Flow cytometry and IF results showed that overexpression of P4HA2 reversed the effect of KynA on the reduction of lipid droplets accumulation in colon cancer cells, while overexpression of P4HA2 lost its reversal effect when HILPDA was knocked down. **D.** The expreesion of ATGL, E-cadherin, N-cadherin, Vimentin, Cyclin E1 and Cyclin D1 in colon cancer cells was detected by WB. **E-F.** CCK-8 assays and colony formation assays revealed that overexpression of P4HA2 reversed the KynA-induced inhibition of proliferation in colon cancer cells, while overexpression of P4HA2 lost its reversal effect when HILPDA was knocked down. **G-H.** The transwell assays and wound healing assays showed that overexpression of P4HA2 reversed the KynA-induced weakened migrative ability of colon cancer cells, while overexpression of P4HA2 lost its reversal effect when HILPDA was knocked down. All data were revealed as mean ± standard deviation for no less than three independent experiments. Significant P values showed as ∗∗∗∗*P* < 0.0001. ∗∗∗*P* < 0.001. ∗∗*P* < 0.01. ∗*P* < 0.05.

We further validated *in vitro* that the KynA/P4HA2/HILPDA axis mediates lipid metabolism reprogramming in tumor cells. ELISA, flow cytometry, IF, and WB results suggested that P4HA2 overexpression reverses KynA’s effect on increasing ATGL enzyme activity and reducing lipid droplet accumulation. However, this reversal effect of P4HA2 was diminished when HILPDA was knocked down (Figure 8A–D, Fig. S4D and E). Similarly, the impact of the KynA/P4HA2/HILPDA axis on colon cancer cell proliferation and migration was confirmed. P4HA2 overexpression reversed KynA’s inhibitory effects on colon cancer cell proliferation and migration. However, this effect was nullified when HILPDA was knocked down (Figure 8D–H, Fig. S4F-H).

**Fig. S5:**
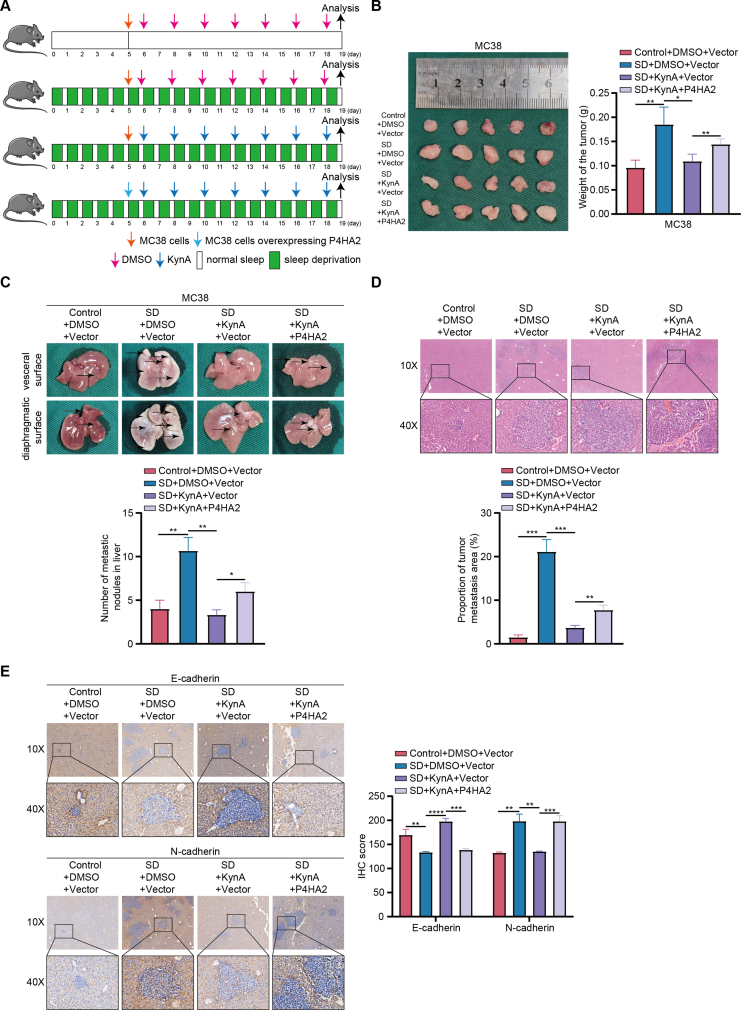
Sleep deprivation promotes colon cancer proliferation and liver metastasis via KynA/P4HA2/VHL/HIF-1α/HILPDA axis. **A.** Schematic illustration of experiment design of a mouse model of sleep deprivation. DMSO or KynA intraperitoneal injection every two days. **B.** KynA treatment reversed the proliferative effect of sleep deprivation on colon cancer, while KynA lost its reversal effect when P4HA2 was overexpressed. Subcutaneous xenografts were peeled off and the weight was measured. **C.** KynA treatment reversed the prometastatic effect of sleep deprivation on colon cancer, while KynA lost its reversal effect when P4HA2 was overexpressed. **D.** HE staining showed the tumors in the live of mice and the percentage of infiltrated area of liver metastases. **E.** The expression level of E-cadherin and N-cadherin was detected by IHC in liver metastases. All data were revealed as mean ± standard deviation for no less than three independent experiments. Significant P values showed as ∗∗∗∗*P* < 0.0001. ∗∗∗*P* < 0.001. ∗∗*P* < 0.01. ∗*P* < 0.05.

Subsequently, we assessed the impact of the KynA/P4HA2 axis on colon cancer proliferation and liver metastasis in mice subjected to sleep deprivation. Following the construction of the mouse sleep deprivation model, we established both subcutaneous xenograft and the liver metastasis models (Fig. S5A). Consistent with the previous results, KynA (100 μL, 10 mg/kg) treatment reduced the size and weight of subcutaneous xenografts compared to the sleep-deprived group. However, colon cancer cells overexpressing P4HA2 showed resistance to KynA treatment, increasing in xenograft size and weight (Fig. S5B). The average tumor weight in Control + DMSO + Vector group, SD + DMSO + Vector group, SD + KynA + Vector group, and SD + KynA + P4HA2 group was 0.10 ± 0.02 g, 0.19 ± 0.04 g, 0.11 ± 0.01 g, and 0.14 ± 0.01 g. In the liver metastasis model, P4HA2 overexpression also counteracted the anti-metastatic effect of KynA, leading to an increased number of liver metastatic nodules and a larger infiltrating area (Fig. S5C and D). The average infiltration area in Control + DMSO + Vector group, SD + DMSO + Vector group, SD + KynA + Vector group, and SD + KynA + P4HA2 group was 1.51 ± 0.52 %, 21.13 ± 2.78 %, 3.72 ± 0.53 %, and 7.77 ± 1.10 %. IHC analysis further demonstrated that KynA mitigated the effects of sleep deprivation on enhancing colon cancer metastasis. However, this rescue effect was impaired in the presence of P4HA2 overexpression (Fig. S5E).

In conclusion, our results validate the role of the KynA/P4HA2/VHL/HIF-1α/HILPDA axis in promoting colon cancer progression due to sleep deprivation.

### 3.8 The P4HA2/HIF-1α/HILPDA signaling axis on colon cancer

**Fig. S6:**
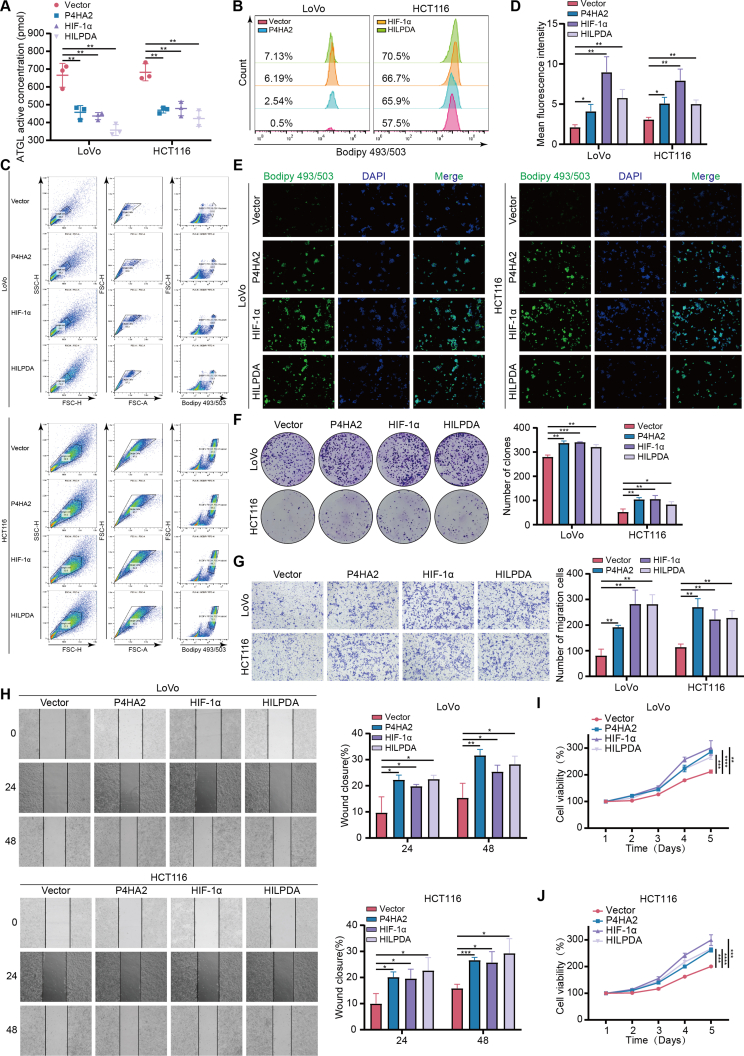
The P4HA2/HIF-1α/HILPDA signaling axis on colon cancer. **A.** ELISA assays showed that overexpression of P4HA2, HIF-1α and HILPDA decreased the ATGL enzyme activity in colon cancer cells. **B-E.** Flow cytometry and IF results showed that overexpression of P4HA2, HIF-1α and HILPDA increased lipid droplets accumulation in colon cancer cells. **F.** Colony formation assays revealed that overexpression of P4HA2, HIF-1α and HILPDA promoted proliferation of colon cancer cells. **G-H.** The transwell assays and wound healing assays showed that overexpression of P4HA2, HIF-1α and HILPDA enhanced migrative ability of colon cancer cells. **I-J.** CCK-8 assays revealed that overexpression of P4HA2, HIF-1α and HILPDA promoted proliferation of colon cancer cells. All data were revealed as mean ± standard deviation for no less than three independent experiments. Significant P values showed as ∗∗∗∗*P* < 0.0001. ∗∗∗*P* < 0.001. ∗∗*P* < 0.01. ∗*P* < 0.05.

We explored the effects of P4HA2, HIF-1α, and HILPDA in colon cancer cell lines, the results suggested that overexpression of P4HA2, HIF-1α, and HILPDA promoted lipid droplet accumulation in colon cancer cells by reducing ATGL enzyme activity (Figs. S6A–E) and promoted the proliferation and migration of colon cancer cells (Figs. S6F–J).

**Fig. S7:**
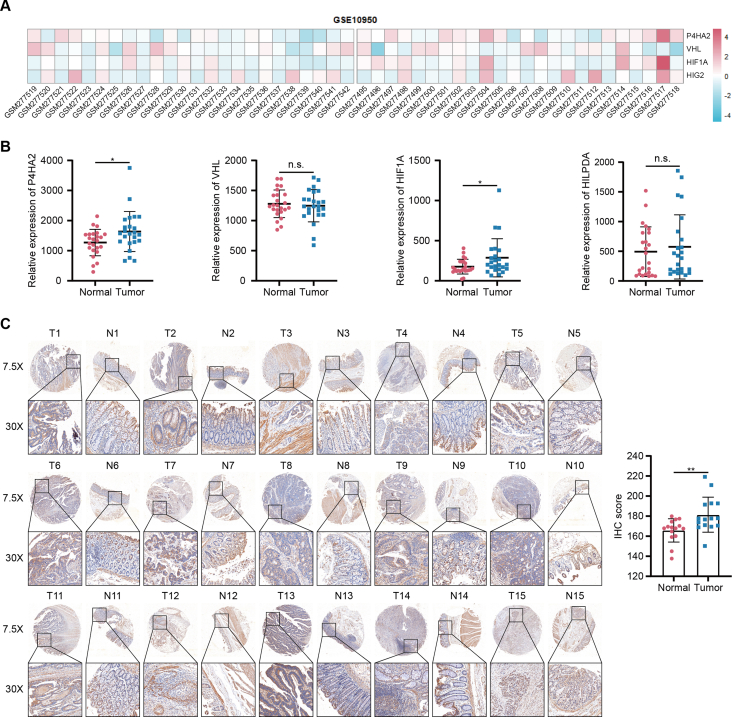
The P4HA2/HIF-1α/HILPDA signaling axis on colon cancer. **A-B.** Relative expression of P4HA2, VHL, HIF-1α and HILPDA in 24 paired samples from GSE database. **C.** The expression level of P4HA2 was detected by IHC in tissue chip. Significant P values showed as ∗∗*P* < 0.01. ∗*P* < 0.05. n.s. means the difference was not significant.

Furthermore, we used GEO dataset GSE10950 and tissue chips to assess P4HA2/VHL/HIF-1α/HILPDA signaling axis on colon cancer. The analysis of GSE10950 showed that the expression of P4HA2 and HIF-1α in cancer tissues was higher than that in adjacent tissues, although there was no difference in the expression of VHL and HILPDA (Fig. S7A and B). Similarly, IHC staining of tissue chips showed that the expression of P4HA2 in cancer tissues was higher than that in adjacent tissues (Fig. S7C).

In conclusion, our results validate the role of the P4HA2/HIF-1α/HILPDA signaling axis in promoting colon cancer progression.

## 4 Discussion

Sleep plays a critical role in overall health. Insufficient sleep has been linked to various health risks, including type 2 diabetes (relative risk range 1.09–1.40), coronary artery disease (relative risk 1.23), stroke (relative risk 1.05), and cancer (relative risk range 1.01–1.32) [4,37]. There is growing evidence that sleep deprivation enhances cancer cell proliferation and metastasis, as well as immune evasion [5,38,39]. Lack of sleep not only fosters tumor development but can also be magnified by cancer, which often disrupts sleep due to pain and associated treatment side effects [3]. Additionally, cancer cells secrete various cytokines that affect the brain through the humoral pathway and activate microglia, altering behavior and sleep patterns [40,41]. Therefore, there is an inseparable positive feedback regulatory loop between sleep disturbances and cancer, suggesting that interventions targeting sleep could potentially treat cancer outcomes.

The brain-gut-microbiome axis represents a crucial communication pathway between the central nervous system and the peripheral systems. The gut microbiota plays a significant role in linking sleep disturbances with intestinal disease. Evidence indicates that sleep disruptions can disrupt the circadian rhythms of gut microbes, affecting their composition and function [42,43]. Conversely, the gut microbiota can respond to sleep disorders [44] and mediate intestinal diseases, such as intestinal inflammatory bowel diseases [45] and intestinal tumors [46].

Metabolites produced by the gut microbiome are central to these interactions. Sleep disorders are associated with elevated levels of short-chain fatty acids in the gut, which can lead to intestinal inflammation [[47], [48], [49]]. Zhao et al. reported that sleep deprivation reduced GABA, a metabolite of the intestinal microbiota in monkeys, and that lactobacilli supplementation could alleviate GABA contents in intestinal inflammation [31]. These findings suggest that sleep deprivation might amplify intestinal diseases, such as colon cancer, by affecting the gut microbiota metabolism and metabolite levels.

Our study found that sleep deprivation enhanced colon cancer proliferation and liver metastases. Notably, the results of untargeted metabolomics revealed disturbed gut microbiota metabolite profiles following sleep deprivation. We specifically focused on KynA, a tryptophan metabolite whose levels significantly decreased with sleep deprivation. Metabolism gene EC: 1.13.11.11, a microbiota rich in tryptophan, has been reported to work symbiotically with intestinal cells to promote the biosynthesis of KynA and reduce inflammation in the body [16]. Ye et al. reported that KynA could improve the efficacy of human adipose mesenchymal stem cells in the treatment of colonic fibrosis in Crohn’s disease [50]. In contrast, Walczak et al. also found that KynA could inhibit the proliferation and DNA synthesis of colon cancer *in vitro* [23,51]. However, the specific mechanisms remain unclear. Our *in vitro* results confirmed that KynA inhibits colon cancer cell proliferation and migration. KynA treatment also mitigated the effects of sleep deprivation on tumor growth and liver metastasis in mice.

Circadian rhythm disruptions caused by sleep deprivation affect various metabolic processes, including glucose, glutamate, bile acids, and lipid metabolism [52]. Lipid metabolism disorders are particularly prominent in cancer, where altered lipid metabolism supports tumor proliferation, survival, migration, invasion, and metastasis [25]. Peng et al. reported that sleep deprivation disrupts circadian rhythms and maintains sleep deprivation, enhancing cancer cell stemness by disrupting cellular lipid metabolism and interfering with fatty acid oxidation in tumor cells [53]. Further evidence recognizes that lipid droplet accumulation is a hallmark of hypoxic cancer cells [26]. In our study, oil red O staining of subcutaneous xenografts and liver metastases in mice revealed that sleep deprivation increased lipid droplet accumulation in colon cancer, which KynA treatment could reverse.

Various evidence also suggests that lipid droplet accumulation often results from inhibited intracellular lipolysis, with ATGL being a key player promoting cancer development [54,55]. HILPDA is known to inhibit ATGL activity [56] and promote lipid droplet accumulation [57,58]. Notably, HIF-1α has been shown to bind to the promoter region of the HILPDA gene to activate transcription of HILPDA [59], as well as promote tumor proliferation, metastasis, and drug resistance [[60], [61], [62]]. Chen et al. reported that deubiquitination of HIF-1α promotes fatty acid synthesis and, therefore, cholangiocarcinoma progression [61]. Our study found that KynA increased the ATGL enzymatic activity and reduced lipid droplet accumulation. However, this effect was reversed by HILPDA overexpression or supplementation with exogenous PA, suggesting that HIF-1α mediated HILPDA expression contributes to lipid droplet accumulation and tumor development *in vivo*.

Hypoxia-inducible factor (HIF-1) is an oxygen-dependent activator of transcription, of which HIF-1α is one of its subunits [63]. The ubiquitin-proteasome pathway mediates the most influential molecular mechanism known to affect HIF-1α activity [64]. Lu et al. reported that ubiquitinated degradation of HIF-1α reduced IL-1β production, which in turn attenuated inflammation and metabolic disorders [65]. Shi et al. reported that CDC20 promoted the progression of liver cancer by inhibiting the HIF-1α ubiquitination [66]. Our results discovered that KynA reduces HIF-1α protein stability and promotes its degradation through the ubiquitin-proteasome pathway, thereby inhibiting the proliferation and liver metastasis of colon cancer.

Collagen prolyl 4-hydroxylase(P4H) is a dioxygenase that catalyzes 4-hydroxylation of proline [67], although it has been reported that P4HA2 inhibits the growth of lung cancer by enhancing mTOR stability [68]. However, in most malignancies such as bladder cancer, ovarian cancer, and B-cell lymphoma, tumors with high expression of P4HA2 have been observed to have a higher degree of malignancy [34,69,70]. Li et al. also reported that P4HA2 enhanced HIF-1α protein stability [34]. Our findings showed that KynA down-regulated P4HA2 expression levels and P4HA2 mediated the regulation of KynA’s on HIF-1α protein stability and ubiquitination modification. P4HA2 knockdown decreased HIF-1α protein stability, while its overexpression of P4HA2 reversed KynA’s effect on promoting degradation of HIF-1α protein.

E3 ligase is an essential component of the ubiquitination process. Numerous studies have shown that VHL is an E3 ligase that mediates HIF-1α protein ubiquitination, and the reduction of its degradation, in turn, is caused by the VHL deficiency promoting cancer progression [[71], [72], [73]]. In our study, we confirmed that P4HA2 regulates HIF-1α protein stability and ubiquitination modification through VHL-mediated and that both KynA treatment and transfection of P4HA2 siRNA diminished the regulatory effect upon VHL knockdown. Additionally, E3 ligases, such as FBXW7, are activated to auto-ubiquitination [36]. Similarly, we discovered that P4HA2 promoted ubiquitination degradation of VHL proteins. Therefore, our study suggested that KynA downregulates P4HA2, inhibiting VHL ubiquitination degradation, and thereby affecting HIF-1α protein stability and degradation.

## 5 Conclusions

**Figure 9:**
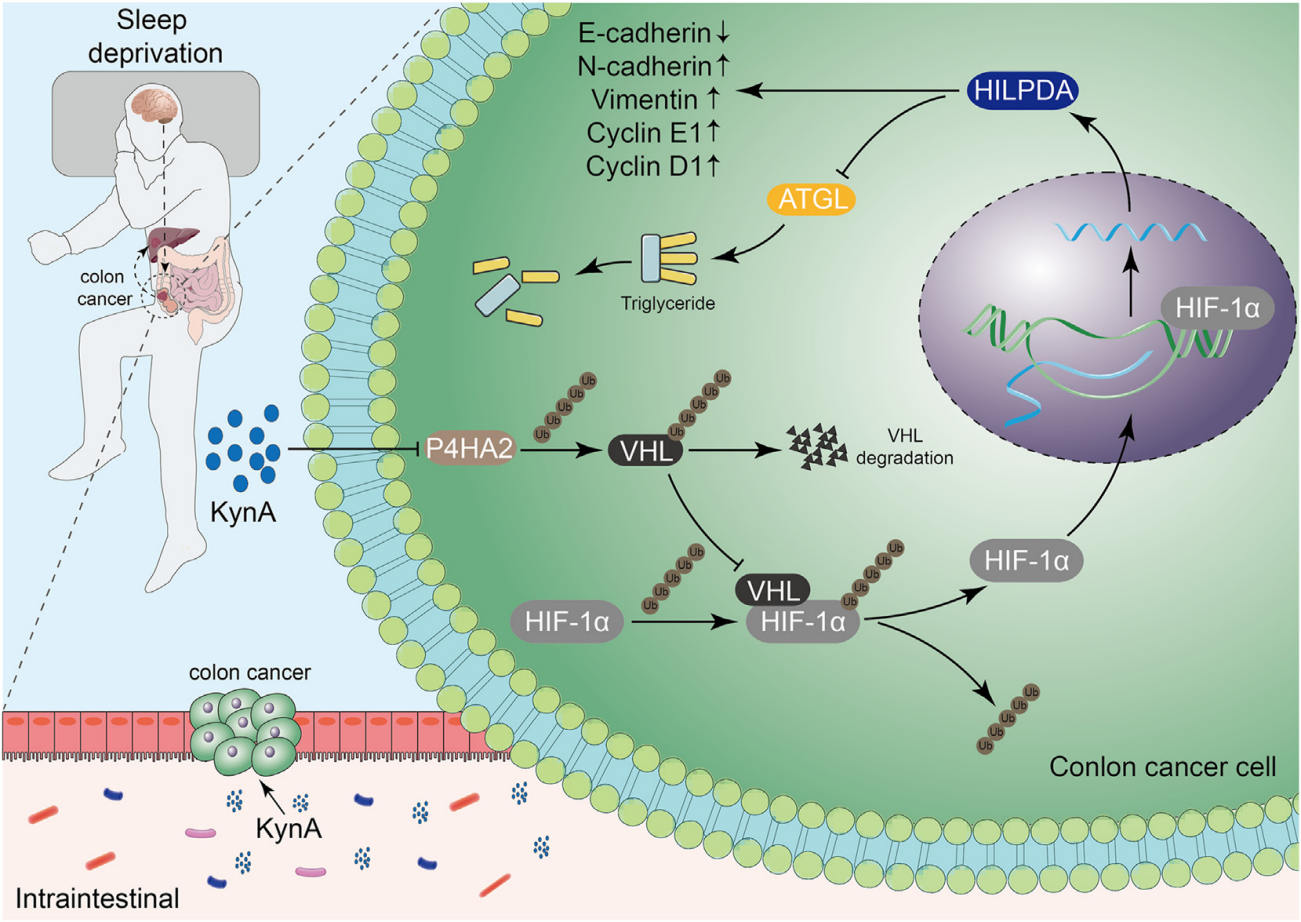
The schematic diagram demonstrated that sleep deprivation promotes colon cancer progression through the gut microbiota metabolite KynA-mediated tumor cell lipid metabolism reprogramming.

In conclusion, our study highlights a novel mechanism by which sleep deprivation promotes colon cancer progression through gut microbiota metabolite KynA-mediated tumor cell lipid metabolism reprogramming (Figure 9). We demonstrate that P4HA2 regulates the ubiquitination and degradation of HIF-1α by endogenously modulating VHL ubiquitination and degradation. Additionally, HIF-1α acts as a transcription factor that regulates HILPDA, leading to reduced ATGL enzymatic activity and increased lipid droplet accumulation. This accumulation, in turn, enhances colon cancer cell proliferation and migration.

Ensuring adequate sleep remains crucial in overcoming cancer. Nonetheless, the complex relationship between sleep and tumor progression requires further exploration. Our study suggests that exogenous supplementation of the gut microbiota metabolite KynA may offer a potential therapeutic strategy for colon cancer treatment and slowing tumor progression. However, further research is needed to validate KynA’s efficacy in human clinical settings.

## CRediT authorship contribution statement

**Zuojie Peng:** Writing – original draft, Software, Project administration, Methodology, Formal analysis, Data curation, Conceptualization. **Jia Song:** Writing – review & editing, Software, Methodology, Formal analysis, Conceptualization. **Wenzhong Zhu:** Writing – review & editing, Software, Formal analysis, Conceptualization. **Haijun Bao:** Writing – review & editing, Software, Formal analysis, Conceptualization. **Yuan Hu:** Writing – review & editing, Software, Methodology, Formal analysis. **Yongping Shi:** Writing – review & editing, Software, Methodology, Formal analysis. **Xukai Cheng:** Writing – review & editing, Software, Methodology, Formal analysis. **Mi Jiang:** Software, Investigation. **Feifei Fang:** Software, Investigation. **Jinhuang Chen:** Writing – review & editing, Visualization, Supervision, Resources, Project administration, Funding acquisition, Conceptualization. **Xiaogang Shu:** Writing – review & editing, Visualization, Validation, Supervision, Resources, Project administration, Funding acquisition, Conceptualization.

## Declarations

All animal experiments were conducted in accordance with the regulations of the Institutional Animal Care and Use Committee of Huazhong University of Science and Technology (IACUC Number: 3982). All contributing authors agreed to the publication of this article and announced no conflicts of interest. This study was supported by the National Natural Science Foundation of China (Grant number: 82072744; 822074874).

## Declaration of competing interest

We declare that we have no financial and personal relationships with other people or organizations that can inappropriately influence our work, there is no professional or other personal interest of any nature or kind in any product, service and/or company that could be construed as influencing the position presented in, or the review of, the manuscript entitled, “Impact of Sleep Deprivation on Colon Cancer: Unraveling the KynA-P4HA2-HIF-1α Axis in Tumor Lipid Metabolism and Metastasis”.

This manuscript describes original work and has not been published in whole or in part nor is it being considered for publication elsewhere. In addition, all authors have approved the manuscript for submission and without any potential competing interests.

## Data Availability

Data will be made available on request.

## Abbreviations

SD: sleep deprivation
KynA: kynurenic acid
DMSO: dimethyl sulfoxide
CHX: cycloheximide
P4HA2: prolyl 4-hydroxylase subunit alpha 2
VHL: von Hippel-Lindau tumor suppressor
HIF-1α: hypoxia inducible factor 1 alpha
HILPDA: hypoxia inducible lipid droplet associated
ATGL: adipose triglyceride lipase
IF: immunofluorescence
ELISA: enzyme linked immunosorbent assay
ChIP: chromatin immunoprecipitation
WB: western blot
